# Determining Dietary Patterns to Recommend for Type 2 Diabetes: An Umbrella Review

**DOI:** 10.3390/nu15040861

**Published:** 2023-02-08

**Authors:** Cathryn Whiteley, Fiona Benton, Louisa Matwiejczyk, Natalie Luscombe-Marsh

**Affiliations:** 1 Research and Program Development, Diabetes SA, Hilton, SA 5033, Australia; 2 College of Nursing and Health Sciences, Flinders University, Adelaide, SA 5042, Australia

**Keywords:** dietary patterns, type 2 diabetes, umbrella review, glycaemia, adults

## Abstract

Some specific dietary patterns improve glycaemic levels and cardiovascular risk factors better than others. We aimed to identify the most effective dietary patterns using a food-focused approach to improve blood glucose management (primary outcome) and cardiovascular risk factors (secondary outcome) in people with type 2 diabetes. An umbrella review was conducted comparing dietary patterns for the management of these outcomes. Studies published between 2012 and 2022 were identified using PubMed Central, ProQuest, Web of Science, and the Cochrane Database of Systematic Reviews. Thirty systematic reviews met the inclusion criteria. Twenty-two of thirty reviews quantitated (via meta-analyses of over 212 randomised control trials) the effect size of different dietary patterns. Twelve reviews found Low-carbohydrate (LC), Mediterranean (M), Plant-based (PB), and/or Low-glycaemic Index (LGI) diets reduced HbA1c moderately more than control diets (typically a high-carbohydrate, low-fat diet) (i.e., LC: −0.1 to −0.5%; M: −0.3 to −0.5%; PB: −0.2 to −0.4%; LGI −0.2 to −0.5%; all *p*-value < 0.01). We conclude that Low-carbohydrate, Mediterranean, Plant-based, and Low-glycaemic Index dietary patterns are all clinically effective for people with type 2 diabetes as alternatives to high-carbohydrate, low-fat diets typically used for managing glycaemic levels and CVD risk. However, quality evidence about the sustainability of effects and safety remains limited, warranting future research.

## 1. Introduction

Type 2 diabetes is characterised by chronic raised blood glucose levels due to a lack of insulin produced by the pancreas or ineffective use of the insulin produced. The condition has nearly doubled since 1980 and is a major public health issue worldwide [1]. Globally, an estimated 462 million individuals are living with diabetes; in Australia, over 1.4 million people have been diagnosed with diabetes [1,2]. Diabetes is a primary cause of blindness, kidney failure, limb amputations, heart conditions, and reduced quality of life. Adults with diabetes have a two- to three-fold increased risk of heart attacks and strokes and an increased risk of premature death [1]. Type 2 diabetes is the most common type of diabetes, representing approximately 95% of all cases of diabetes globally and 89% of cases in Australia [3]. It is recognised as a partially preventable condition that can be treated with a healthy diet, regular physical activity, and weight management [1].

Although regulating dietary factors is critical to effectively managing diabetes, an initial scoping of Australian and International nutrition guidelines for people with diabetes identified a lack of consensus over what to recommend [3,4]. Moreover, navigating the plethora of public nutrition messages is complicated and confusing for people living with type 2 diabetes and the general practitioners who are the frontline primary health professionals people turn to first for support [5]. More than 20% of Australians with type 2 diabetes also have cardiovascular disease (CVD), and a smaller percentage also live with other chronic conditions [3]. These people and their supporting health professionals need (and want) clear, evidence-based dietary recommendations that are relevant to them and can easily be implemented into their everyday life [2,6,7].

The population approach to supporting people with type 2 diabetes follows the Australian National Dietary Guidelines designed for the general population (high carbohydrate, low fat with 45–65% total energy intake from carbohydrates and 20–35% of total energy intake from fat). However, it is well established that compliance with the national dietary guidelines is poor, with more than 50% of Australians not meeting the recommendations highlighted in recent food consumption data [8]. To emphasise this, only 5.4% of adults meet both fruit and vegetable recommendations [9], while 35% of adults’ daily energy intake was from discretionary food choices [8]. Discretionary foods are foods and drinks which are high in saturated fats, sugar, salt, or alcohol and as such, are energy-dense with relatively few nutrients that the body needs [10]. Moreover, while many national dietary guidelines are based on healthy food choices, the proportions of recommended foods are not designed to support diabetes and other chronic conditions [7].

Ongoing research suggests that an approach that brings about sustained dietary management can lead to haemoglobin A1c (HbA1c) and cardiovascular disease (CVD) risk reduction over an extended period [7,11,12]. Furthermore, sustaining a healthy body weight is an important factor in the effective management of type 2 diabetes. However, diets that promote the active restriction of certain foods to achieve weight loss can negatively impact, stigmatise or undermine a person’s positive relationship with food and ability to sustain a healthy lifestyle [13,14]. Of particular interest for this umbrella review are previous reviews that have tried to isolate the effect of dietary patterns from energy restriction or individualised changes usually made during medical nutrition therapy [15,16,17]. However, people with type 2 diabetes often find that nutrition-based treatment provided by an accredited dietitian is not always accessible, affordable, or suitable due to numerous barriers described in studies [18,19].

Over the last decade, there has been an increasing number of dietary approaches for supporting people with type 2 diabetes. Alternative dietary approaches to national guidelines focused on dietary patterns include Dietary Approaches to Stop Hypertension (DASH), Intermittent fasting, Low-carbohydrate, Low-fat, Low-glycaemic Index, Mediterranean and Plant-based options. Collating the latest evidence from a number of systematic reviews that have examined various eating patterns should help alleviate confusion about which dietary patterns are optimal for type 2 diabetes management [7,20]. Therefore, this umbrella review aims to identify the most effective dietary pattern delivering a food-focused approach to improve blood glucose management and CVD risk for people with type 2 diabetes.

## 2. Materials and Methods

An initial scoping search was conducted to identify any form of review that had examined specific dietary approaches to improve blood glucose levels in people with diabetes without a focus on weight loss or medical nutrition therapy, and also national and international consensus reports and articles [6,17,21,22,23]. Several extensively researched dietary patterns [23] have effectively reduced glycaemic levels and cardiovascular risk factors [24,25] and the background information for seven commonly researched dietary patterns is depicted in Table 1. Namely, (1) Dietary Approaches to Stop Hypertension (DASH), (2) Intermittent Fasting, (3) Low-carbohydrate (or Ketogenic or High protein or Paleo), (4) Low fat, high carbohydrate, (5) Low-glycaemic Index (or High fibre or Prudent), (6) Mediterranean (or MUFA or Nordic) and (7) Vegetarian (or Vegan or Portfolio or Flexitarian). The scoping review determined the search terms used for the final search, the databases to be searched, and the inclusion and exclusion criteria to determine which full-text articles would be included in the evidence synthesis (Table 2). The databases were searched for relevant reviews published between 2012 and 2022. The titles and abstracts of all returned reviews were examined independently by one of two reviewers (CW, IM) against the inclusion and exclusion criteria to determine if they were suitable to move to the full-text screening stage. Additionally, 10% of the title and abstracts were reviewed by both reviewers, and in the case of any discrepancy, a third reviewer (NLM) was consulted for consensus. The full text of reviews that made it through the title and abstract screening stage were then examined against the eligibility criteria for inclusion into the synthesis of evidence (reviewed by CW and IM with NLM consulted for consensus). Endnote aided collation of the systematic review papers identified from the different databases. The reference lists were checked for additional reviews that were missed, and duplicates were removed prior to at least one reviewer (CW or IM) screening all remaining full-text reviews to exclude those not meeting the inclusion criteria.

Full-text reviews identified from the screening process were attained, and a quality appraisal was conducted on each paper by one of two independent reviewers (CW, IM). To ensure some consistency between the two reviewers doing the quality appraisal, 10% of the full-text articles were reviewed by both reviewers. In the case of any discrepancy, a third reviewer (content expert NLM) was consulted for consensus. The quality appraisal of the reviews was based on information narratively reported with each systematic review. In addition, the Cochrane Collaboration assessment tool was used to rate each systematic review for bias types categorized into one of six domains–i.e., selection bias (random sequence generation and allocation concealment), performance bias (blinding of participants and personnel), detection bias (blinding of outcomes assessment), attrition bias (incomplete outcome data), reporting bias (selective reporting), and other biases (e.g., no consideration given to potential confounders or publication bias) [47]. Each full-text review included in this umbrella review was classified as being at a low risk of bias if, across the review, three or more of the six domains of bias were rated as low risk; at a high risk of bias if two or more of the six domains were rated as high risk across the review; or at an unclear risk of bias if three or more of the six domains were rated as unclear or not rated at all across the review [48].

Following the quality and risk appraisals, the relevant data of interest were extracted from each selected review following the PICO strategy (Table 2); more specifically, this included the author/year, country of origin, the total number of studies included, participants (total number/ages), objectives, duration of included studies, information about the intervention and control diets, and information about the review outcomes (i.e., effect size of the mean difference between the intervention and control diets for HbA1c, FBG, FBI, weight, and the statistical significance/direction of the change).

## 3. Results

### 3.1. Identification of Records Included in This Umbrella Review

**Figure 1:**
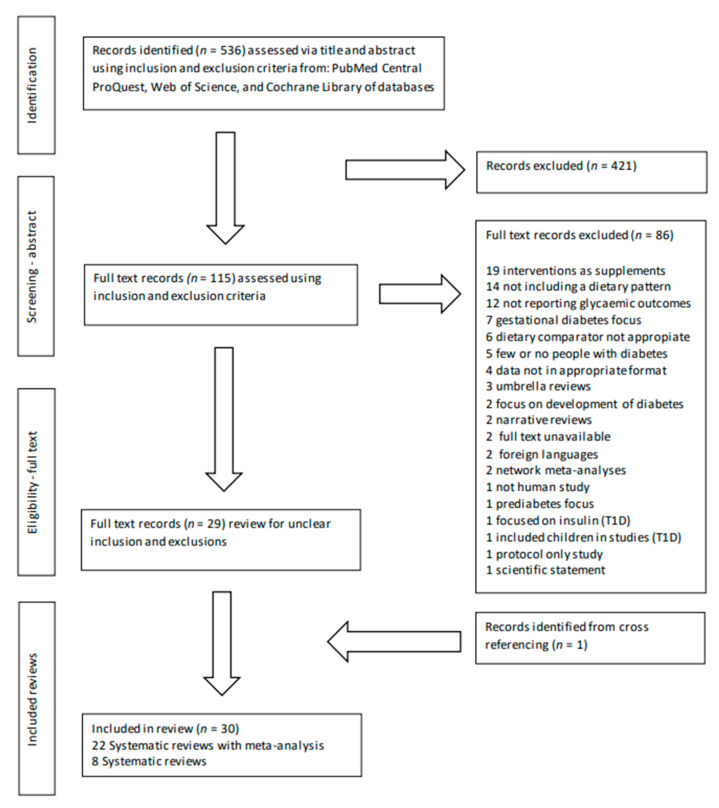
Review selection PRISMA flow diagram.

Five hundred and thirty-six articles were identified from the search strategy. The PRISMA flowchart (Figure 1) summarises the number of articles included and excluded at each stage of the screening process. Following the title and abstract screening stage, 115 articles underwent the full-text screening, from which 86 records were excluded because they did not fulfil the inclusion criteria (details of excluded articles can be found in Supplementary Table S1). Therefore, a total of 29 articles that meet all the inclusion criteria proceeded to the data extraction phase of this umbrella review. During data extraction, the reference lists of the included articles were checked, and one additional article was identified for inclusion in the umbrella review. The total number of articles extracted for inclusion was 30 articles: 22 systematic reviews with meta-analysis and 8 without meta-analysis.

No systematic reviews on the effects of Dietary Approaches to Stop Hypertension (DASH) or Intermittent Fasting diet outcomes related to glycaemic management met the inclusion criteria for this umbrella review. Low fat or High carbohydrate diets within the publication date revealed that these diets were commonly used as the control diet or comparator intervention rather than the dietary pattern under investigation. The dietary patterns that represented the patterns for investigation included: multiple intervention diets [31,34,49,50], Low-carbohydrate [27,28,44,45,51,52,53,54,55,56,57,58,59,60,61,62], Mediterranean [32,33,63,64,65], Plant-based [66,67,68,69,70], and Low-glycaemic Index [39,71] diets.

Three quarters of the included review articles were systematic reviews with meta-analysis [27,32,33,34,39,44,45,51,52,53,54,55,56,57,58,61,62,63,66,67,68,71], and one-quarter were systematic reviews [28,31,49,50,59,65,69,70]. Thirty systematic reviews (twenty-two with meta-analysis and eight without) were sourced in this review, with twenty-seven compiling their reviews about dietary patterns for people with type 2 diabetes. However, three systematic reviews with meta-analysis examined randomised control trials and had a mixture of adults with type 1 and type 2 diabetes. Since the greater proportion of the overall study population was people with type 2 diabetes, we included these three systematic reviews in this umbrella review [66,67,71].

### 3.2. Characteristics of the Systematic Reviews Included in This Umbrella Review

The study characteristics for 30 systematic reviews that met all inclusion and exclusion criteria are reported in Supplementary Table S2. The included systematic reviews each examined between 6 and 36 randomised control trials of dietary interventions undertaken across various countries. Most randomised controlled trials collectively occurred in the United States (*n* = 59), with the next highest number from Australia (*n* = 30), followed by the United Kingdom (*n* = 19). Further, countries with larger numbers of randomised control trials or multiple reviews undertaken included Israel (*n* = 14), Japan (*n* = 14), Sweden (*n* = 13), and Canada (*n* = 9). The age range across 30 systematic reviews reporting on type 2 diabetes ranged from 30 years to 82 years of age. The duration of studies within the systematic reviews ranged from 1 week to 224 weeks. Of the 30 reviews with and without meta-analyses that were examined, 17 contained individual studies whose duration was at least 12 to 224 weeks. In contrast, for the other 13 reviews, the durations for the studies were between 1 to 103 weeks.

The selected systematic reviews investigated were grouped to enable comparisons under overarching dietary pattern categories; Low-carbohydrate (*n* = 18), Mediterranean (*n* = 8), Plant-based (*n* = 8), Low-glycaemic Index (*n* = 5), including dispersed multiple dietary pattern reviews. Four systematic reviews present the effect of multiple different dietary patterns and their control diets on the management of type 2 diabetes [31,34,49,50]. One multiple dietary pattern review by Ajala et al. [34] includes a meta-analysis; therefore, the quantitative results have been included and separated into three corresponding dietary pattern categories. A high-carbohydrate, low-fat dietary pattern was the main control diet reported in the systematic reviews included in this umbrella review, which is a dietary pattern consistent with many national dietary guidelines [10].

For the remainder of the results section, only the results from the 22 systematic reviews with meta-analysis will be reported because they quantitatively described each dietary pattern’s effect size on the outcomes. However, the results from the additional eight systematic reviews without meta-analysis are reported within the supplementary tables. They will be used to inform the discussion because they provide additional support to the findings derived from the systematic reviews with meta-analyses. Table 3 depicts the systematic reviews with and without a meta-analysis.

Approximately 29,590 participants were included across the 22 systematic reviews with meta-analysis including 19,112 undertaking a Low-carbohydrate diet, 6875 a Mediterranean diet, 1117 Plant-based diet, and 2486 Low GI diets.

### 3.3. Primary Outcomes

The primary outcomes of HbA1c and fasting blood glucose (FBG) for the 22 systematic reviews with meta-analysis relating to each dietary pattern are summarised in Table 4. The data are presented under four overarching dietary pattern categories: Low-carbohydrate, Mediterranean, Plant-based, and Low-glycaemic Index. The results for all systematic reviews with and without meta-analyses are provided in Supplementary Tables S2 to S8.

Six of fourteen systematic reviews with meta-analysis investigating Low-carbohydrate diets indicated statistically greater mean reductions in HbA1c for up to 12 months, with the mean difference between the Low-carbohydrate diets and the control diets ranging from 0.1 to 0.5% [34,45,51,54,61,62]. An additional four reviews also reported the mean differences in HbA1c reductions were greater and of similar magnitude as reported above with the Low-carbohydrate diet, but statistical significance was not reached [44,52,56,57]. Concerning fasting blood glucose, 5 of the 14 systematic reviews with meta-analysis reported no difference between dietary patterns [27,44,54,58,61]. One review examining fasting blood insulin revealed no difference between intervention and control diets [27].

In three of four systematic reviews with meta-analysis, Mediterranean diets compared to control dietary patterns resulted in significantly greater mean reductions in HbA1c, with the mean difference between diets ranging from 0.3 to 0.5% [32,34,63]. In addition, the multiple dietary pattern review, which compared either Low-carbohydrate, Mediterranean or Low-glycaemic Index diets against relevant control diets, reported significant improvements in glycaemic management with all diets [34]. With regard to fasting blood glucose, two of the systematic reviews with meta-analysis also reported significantly greater reductions following a Mediterranean-style diet (including MUFA) compared to the relevant low-fat control; the mean differences ranged between 0.6 to 0.7 mmol/L [32,33].

Three systematic reviews with meta-analysis of plant-based diets (replacing animal products with plant proteins) reported statistically greater improvements in glycaemic management following the plant-based than control diets; the mean difference for the reduction in HbA1c ranged from 0.2 to 0.4%. The mean difference for the reduction in fasting blood glucose ranged from 0.5 to 0.6 mmol/L, and the mean difference for the reduction in fasting blood insulin ranged from 1.1 mU/L to 1.5 mU/L [66,67,68].

Low-glycaemic Index dietary patterns were examined in three systematic reviews with meta-analysis, and all showed a statistically greater mean reduction in HbA1c, with the mean difference between the Low than High-glycaemic index diets ranging from 0.2% to 0.5% [34,39,71].

### 3.4. Secondary Outcomes

Systematic reviews with meta-analysis were examined for secondary outcomes, including weight loss and cardiovascular risk factors of blood pressure and lipids; the data are summarised and presented in Table 5. Supplementary Table S8 contains the results for cardiovascular risk factors from both systematic reviews, with and without meta-analyses.

For the Low-carbohydrate dietary patterns, 5 of 14 Low-carbohydrate systematic reviews with meta-analysis reported reductions in weight, with 4 reviews reporting a statistically significant difference of 0.7 to 3.5 kg [34,51,61,62] and 1 reporting non-statistical differences between diets [52]. The other reviews did not report a difference in weight between the two diets [27,34,44,45,51,52,53,54,55,56,57,58]. Blood pressure and lipids were investigated in 12 of the systematic reviews with meta-analyses [34,44,45,51,52,54,55,56,57,58,61,62]. Five reviews reported significantly greater increases in high-density lipoproteins ranging from 0.06 to 0.09 mmol/L [34,45,51,54,62] and four reported reductions in triglycerides of 0.2 to 0.3 mmol/L [45,51,58,62] (Supplementary Table S8). Blood pressure was examined in six of the meta-analyses with no significant reductions [44,45,52,55,56,58,62].

For the Mediterranean-style dietary patterns (including MUFA review), weight was examined in four systematic reviews with meta-analysis [32,33,34,63], with two reviews reporting statistically greater reductions between the two diets, with the mean differences in the reductions ranging from 1.6 to 1.8 kg [33,34] (Supplementary Table S4). The other two reviews reported non-statistical differences between the two diets. Cardiovascular risk factors were investigated in two systematic reviews with meta-analyses of Mediterranean compared to relevant control diets reporting statistically greater improvements in blood pressure and or blood total cholesterol, triglycerides, and high-density lipoprotein cholesterol [32,33].

One of the three plant-based systematic reviews with meta-analysis reported a statistically significant greater reduction in body weight following Plant-based diets compared to animal protein diets [67].

One Low-glycaemic Index review investigated the secondary outcomes of weight and cardiovascular risk factors, including blood pressure and lipids with no statistical differences between dietary patterns [71] and one multiple diet review reported a significant difference for high-density lipoproteins of 0.05 mmol/L in Low-glycaemic Index diets [34].

Assembled below (Table 6) is an ‘at a glance’ overview of the glycaemic and cardiovascular benefits of four food-based dietary patterns researched in this umbrella review.

### 3.5. Quality and Risk of Bias of Appraisals

Quality and risk of bias appraisal tools used in the systematic reviews included within this umbrella review are described in Supplementary Table S9. These included the Cochrane Collaboration handbook and bias risk tool (*n* = 17), the Jadad scale (*n* = 3), AMSTAR quality tool (*n* = 1) along with a review using author critical appraisal skills (*n* = 1).

The quality and risk of bias are summarised in Table 7, and more information is available in Supplementary Table S9.

Approximately 50% of systematic reviews with meta-analysis rated the quality of evidence as moderate-to-high across the four diets [27,32,33,34,39,51,54,61,62,63,66], while 50% rated the overall quality as unclear or low [44,45,52,53,55,56,57,58,65,67,70]. Around 70% of the included systematic reviews with meta-analysis were found to have an unclear risk of bias, 30% had a low risk of bias, while only one review had a high risk of bias. For the reviews with meta-analysis that reported an unclear risk of bias, the main biases were related to selection bias (i.e., random sequence generation and allocation concealment were not carried out appropriately) and performance bias (blinding of participants and personnel). Other limitations regarding the quality and biases present within the systematic reviews mentioned included heterogeneity and missing data, particularly missed data about the adherence to dietary and control interventions.

## 4. Discussion

This umbrella review of 30 previously published systematic reviews (22 with and 8 without meta-analysis) examined different food-focused dietary patterns’ effectiveness in managing type 2 diabetes. The findings show a body of moderate quality evidence with moderate risk of bias that has consistently reported four main dietary patterns as being modestly more effective than high-carbohydrate, low-fat diets typically used in clinical practice. The four patterns are Low-carbohydrate, Mediterranean, Plant-based, or Low-glycaemic Index diets. Although the average difference between these dietary patterns and the control for reductions in blood glucose and lipid levels, blood pressure, and body weight were small, the range of improvements reported may afford a small advantage by positively impacting people living with diabetes. It is well-established that small reductions in HbA1c, both with and without reductions in other cardiometabolic risk factors, and if maintained over the years, assist in reducing the long-term risk of micro- and macro-vascular complications [72,73,74].

This umbrella review showed overlap in the data reported for the minimum and maximum mean differences in HbA1c reductions between the Low-carbohydrate, Mediterranean, Plant-based, and Low-glycaemic Index diets as compared to typical high-carbohydrate, low-fat control diets. Namely, the minimum and maximum mean differences between the dietary comparisons for reductions in HbA1c were −0.1 to 0.5% for Low-carbohydrate, −0.3 to 0.5% for Mediterranean, −0.2 to 0.4% for Plant-based and −0.2 to 0.5% Low-glycaemic Index diets. Fasting blood glucose was not substantially reduced in the reviews with meta-analyses of Low-carbohydrate diets [27,44,54,58], but it was in those on Mediterranean [34,63], Plant-based [65,66], and Low-Glycaemic Index diets [39,70]. Consistent with our findings, a 2019 narrative review [26] and 2018 network meta-analysis [75] both reported that Low-carbohydrate diets and Mediterranean diets were moderately more effective at reducing HbA1c than higher-carbohydrate, low-fat diets [26]. In slight contrast to our findings, a 2018 network meta-analysis by Schwingshackl et al. [48] reported that Low-carbohydrate diets had marginally superior effects on HbA1c alone than Mediterranean diets. However, both Low-carbohydrate diets and Mediterranean diets ranked more highly for reducing HbA1c than Plant-based and Low-glycaemic Index diets. All diets were moderately more effective than higher-carbohydrate, low-fat diets [48].

A 2019 network meta-analysis by Pan et al. [75] also concluded that a Mediterranean diet yielded the most beneficial improvements in blood glucose levels (both HbA1c and fasting blood glucose) for people with type 2 diabetes. These collective findings suggest that the Mediterranean diet may be marginally more effective than any other dietary patterns examined using meta-analysis or network meta-analysis (48, 74). However, there were notably more meta-analyses on Low-carbohydrate diets. Moreover, within all meta-analyses reviewed, nine of fourteen on Low-carbohydrate diets and one of four on Mediterranean diets included individual randomised control studies that ran for more than 52 weeks. In contrast, long-term evidence from randomised control studies on the blood glucose-lowering effects of Plant-based and Low-glycaemic Index diets remains limited. These collective findings, therefore, support the idea that further long-term research is required to reach a consensus on the superiority of any of these four dietary patterns for specifically lowering blood glucose levels in people with type 2 diabetes. It is clear, however, that each of these patterns represent alternative options to the current Australian recommendation of high-carbohydrate, low-fat diets for the population with type 2 diabetes.

The findings for the effects of the four different diets on blood pressure and blood lipids were mixed. Five of the twelve meta-analyses examining the benefits of Low-carbohydrate diets [44,45,51,52,54] and three of the four examining Mediterranean diets [32,33,34] reported small but greater improvements in at least one of the following risk factors compared to a control diet—i.e., systolic blood pressure, triglycerides and total, LDL—and HDL-cholesterol than the comparison control diets. The evidence reported by Kirkpatrick and colleagues 2019 supports our findings that the reductions in cardiometabolic risk factors seen with Low-carbohydrate diets may return towards baseline after two years as people adapt to the pattern during the maintenance phase [26]. The two Mediterranean-style dietary reviews that included cardiovascular risk factors showed various outcomes favouring greater reductions in blood pressure and lipid markers than was observed with a high-carbohydrate, low-fat control pattern [32,33]. A 2019 network meta-analysis by Pan and colleagues regarding the efficiency of multiple diets also indicated that Mediterranean diets resulted in the greatest improvements in cardiovascular risk factors [75]. Cardiovascular risk factors were not always reported in the reviews with meta-analysis on Plant-based and the Low-glycaemic Index diets, but there was evidence from one review that reported greater reductions in LDL-cholesterol with Plant-based than control diets [67]. Additionally, evidence from one review reported greater increases in HDL-cholesterol with Low-glycaemic Index than control diets [34].

With respect to improvements in body weight, the included systematic reviews with meta-analysis reported clinically relevant mean differences with all dietary comparisons that were of similar size. For example, the maximum difference in reduction between the control and the Low-carbohydrate, Mediterranean, and Plant-based, diets, respectively, were −0.7 to −3.5 kg, −1.6 to −1.8 kg, and −2.2 kg. Notably, no clinically relevant difference in body weight reduction was reported with Low-glycaemic Index diets. Although weight loss does not solely cause changes in other cardiometabolic risk factors, previous research in individuals with obesity and type 2 diabetes has demonstrated weight changes as small as 1 kg are associated with small reductions of 0.5–1 mmHg in blood pressure, 0.1% in HbA1c, and 0.02 mmol/L in total cholesterol [76,77]. A recent study on individuals with obesity and type 2 diabetes showed no association between weight loss and changes in plasma lipid despite a 0.6 mmol/mol reduction in HbA1c associated with 1 kg of weight loss [77]. When considered alongside the evidence from the current umbrella review, these associations indicate that multiple mechanisms mediate small changes in blood pressure and lipids.

The mechanisms underpinning the effectiveness of dietary patterns bring together comparable foods from these dietary patterns to guide future principles for this population. This review shares common features of the four dietary patterns identified as being more effective than high-carbohydrate, low-fat diets. These four dietary patterns are all higher in plant foods, including vegetables, legumes, whole grains, fruit, nuts, and unsaturated oils. Predominately, they are lower in animal-based meats and highly processed foods than those usually consumed by individuals following high-carbohydrate, low-fat diets.

Strengths of this review include recent evidence from 22 systematic reviews with meta-analysis from 2012 to 2022 that focus on RCTs and the assessment of the overall quality of the evidence achieved from condensing risk of bias scores from the Cochrane Collaboration tool [47]. Two-thirds of the selected systematic reviews provided moderate quality evidence. The majority of studies included were from the United States of America, Australia, and the United Kingdom, suggesting that these findings are applicable to populations in similar countries. An added strength was that evidence focused on identifying dietary patterns that did not promote energy restriction for weight loss, which can have negative, unintentional impacts, stigmatise, undermine people’s relationship with food and have shown to be unsustainable [12,13]. Furthermore, individualised medical nutrition therapy was not central to implementing the dietary patterns explored in the reported studies. Still, we would expect that the benefits may be greater if these dietary patterns were consumed as part of an energy-restricted diet supervised by a health professional.

A number of limitations must also be acknowledged. One of the greatest difficulties in comparing the benefits of different dietary patterns across the available body of evidence relates to the limited reporting on adherence to the dietary patterns over the short or long term or if people experienced adverse side effects. However, two recent reviews focusing on the efficacy and safety of Low-carbohydrate diets report evidence for mostly the short-term use of Low-carbohydrate diets with a need for monitoring and medication management [61,62]. For the systematic reviews on Low-carbohydrate diets, 9 of the 14 with meta-analysis reported various problems with adherence and attrition (attrition tended to be high when following the diet for more than one year) [44,45,54,55,56,57,58,61]. Two reviews also discussed poorer adherence to Very-Low-carbohydrate diets (i.e., <10% daily carbohydrate intake) [45,52]. Information describing how well people adhere to Mediterranean-style diets, Plant-based diets and Low-glycaemic Index dietary patterns was limited and varied in the reviews where adherence was discussed. A systematic review by Johannesen et al. reported that people changing from omnivorous to Plant-based diets find it challenging to adhere to interventions lasting more than 1 to 2 years [70]. An additional limitation relates to the duplication of data from individual randomised control studies; this was observed across the reviews included in this synthesis which may be seen as a limitation (i.e., evidence is inflated) or strength (i.e., conclusions are aligned). Data from individual studies were also repeated across all Low-carbohydrate systematic reviews, with at least 4 to 10 of the reviews containing the same studies [44,45,52,54,55,56,57,61,62]. This, in turn, suggests that the quantity of evidence for Low-carbohydrate diets may be overstated compared to the other dietary patterns reviewed. However, up to four of the Mediterranean diet reviews also included many of the same major studies [32,33,63,65] and at least two of the Plant-based diet reviews contained the same studies [67,68,69,70].

## 5. Conclusions

A comprehensive review of a body of moderate quality evidence with a moderate risk of bias reveals that Low-carbohydrate, Mediterranean, Plant-based, and Low-glycaemic Index dietary patterns can produce small but clinically relevant improvements in reducing blood glucose levels and at least 1 to 4 common cardiovascular risk factors when compared to high-carbohydrate, low-fat diets. High carbohydrate, low-fat diets are typical of many national dietary guidelines recommended for populations with or without type 2 diabetes, including Australia. Although a high-carbohydrate, low-fat diet is effective, the evidence for the effectiveness of other dietary patterns is accumulating and shows they are all clinically effective alternatives for people managing their type 2 diabetes. The evidence, albeit it is limited, has also shown that people can adopt these dietary patterns for periods of up to 4 years and potentially sustain them for life. As such, these approaches may provide enormous potential for diabetes management and lead to a lowering of subsequent diabetes complications and cardiovascular consequences currently observed in this population. However, quality evidence about the sustainability of effects and safety regarding these patterns remains limited warranting future research.

Maintaining any diet or dietary pattern for life is an ongoing problem for many people. However, regulating food intake plays a major part in improving glycaemic levels for people with type 2 diabetes. Therefore, a dietary pattern should be one that individuals can best sustain. Based on the evidence presented in this umbrella review, the Mediterranean, Plant-based, Low-glycaemic Index, Low-carbohydrate diet, or their shared dietary pattern principles can be recommended for managing type 2 diabetes. These four dietary patterns are all relatively lower in carbohydrate foods than national dietary recommendations for a High-carbohydrate, low-fat dietary pattern, which has poor compliance in Australia and other countries [8]. Introducing options for more than one dietary pattern for the effective management of type 2 diabetes will be helpful for health professionals who support individuals with diabetes as well as those living with the condition. It will also provide people living with this condition greater flexibility to address their food preferences and thereby achieve long-term adherence and success with their diabetes management goals [78]. Policy-makers and program planners can use this study’s findings for consistent, up-to-date, evidence-based recommendations and messaging for people with type 2 diabetes. Future research will continue to evolve new and improved recommendations to ensure guidelines are useful for people with diabetes and replace current ambiguous recommendations and the use of national dietary guidelines for this population.

## Figures and Tables

**Table 1:** Types of dietary patterns identified for supporting the dietary management of Type 2 diabetes.

Dietary Pattern	Description	Foods Included
Low-carbohydrate (LC) diet [26], Very-low carbohydrate diet (VLCD) [26], Paleolithic (paleo) [27], High protein diets (HPD) [28]	LC diet focus on restricting foods high in CHO. There are various levels of CHO restriction–i.e., LC is defined as <26% of total energy (TE) from CHO per day (equates to <130 g/day for an 8360 kJ/day). VLCD is defined as <10% of TE from CHO per day (equates to <50 g/day for an 8360 kJ/day. Paleolithic diets and high protein diets tend to resemble LC because some CHO is replaced by protein.	LC diets promote low-carbohydrate (CHO) vegetables, some low starch fruits and restrict starchy foods, grains, added sugars, lean protein foods, and foods rich in healthy fats and oils. Paleo diets are based on lean meat, fish, fruit, vegetables, eggs, nuts and exclude dairy products, cereal grains, legumes, fats, refined sweets, and sugar. HPD usually replace CHO dense foods with protein and may be low or high in saturated fat.
Mediterranean-style diet (M) [29,30,31,32], including Traditional Mediterranean (TM) and diets high in monounsaturated fatty acids (MUFA) [33]	The M diet reflects diets from the Mediterranean, rich in plant-based foods, olive oil, and fish. TM diets closely follow traditional foods and are used in LC studies as low CHO (LCTM). Energy ranges of 10–55% (TE) from CHO per day, 30–45% (TE) from fat per day (10–50% MUFA) [32,33,34]. High MUFA diets are based on Mediterranean-style eating, including a moderate intake of olive oil (and nuts) and are compared to high CHO control diets.	All emphasise a high intake of unrefined cereals, vegetables, legumes, fruits, nuts, and olive oil, low amounts of saturated fats, a moderately high intake of fish, a low-to-moderate intake of dairy products (cheese or yogurt), eggs and poultry, and a low intake of red meat. M and TM diets include regular but moderate wine consumption with meals.
Plant-based (PB) diet, vegetarian, or vegan diets [34,35,36,37,38]	Plant-based diets focus predominately on eating whole foods from plants, and most exclude meat, poultry, and seafood (or products containing these foods). Energy ranges of 50–78% (TE) from CHO per day, 10–35% fat per day. There are different classifications of Plant-based diets with various inclusions and exclusions and varying daily quantities of foods.	Diets include vegan diets without animal products or bi-products, Lacto includes dairy foods, ovo-lacto contains dairy foods and eggs. Vegetarian diets contain fruits, vegetables (legumes), bread, cereals, dairy products or alternatives, and healthy fats. Semi-vegetarians or flexitarians eat selected animal products sometimes.
Low-glycaemic Index (LGI) [39,40] diet	LGI promotes CHO foods that are less likely to produce significant increases in blood glucose levels. Energy ranges of 37–50% (TE) from CHO per day (GI < 55, 25–42% fat per day. LGI foods are assigned a value as ‘low’ when GI is 55 or less (some studies LGI is assigned 40) compared to high GI foods with a value of 70 or over.	Features include swapping high GI foods for whole, less processed CHO foods with a low–glycaemic index, such as oats, legumes, green vegetables, and other LGI food options from different food groups (i.e., whole grain cereals).
Dietary Approaches to Stop Hypertension (DASH) [41]	The DASH diet is a balanced eating plan limiting sodium, saturated fat, and added sugars. Initially created to lower blood pressure, the approach is used to amend eating habits and reduce cardiovascular disease.	Focuses on intake of fruits, vegetables, low-fat dairy; includes whole grains, poultry, fish, nuts; and supports the reduction in red meat, sweets, sugary beverages, total fat, saturated fat, and cholesterol. Sodium is reduced to 1500–2300 mg/day.
Intermittent fasting diet (IFD) [42,43]	Consists of periods of no and limited calorie intake. Diets generally consist of fasting for 16 h a day, a 24 h period on alternate days, or two days a week.	Comprises of eating less food (restricting); based on omitting food or types of food eaten for certain periods of time.
Low-fat diet (LFD) [44,45,46]	A diet with an overall reduction in fat, reducing total energy to promote health and weight loss. Recently, it has been used as a control or usual diet to compare specific food-based dietary patterns.	Reducing total daily fat intake to approximately 20% of energy (kilocalories) by increasing the daily intake of vegetables, fruits, and grains. Higher daily intake of CHO and protein foods.

**Table 2:** Final inclusion and exclusion criteria, search terms, and PICO strategy used for data extraction.

Inclusion Criteria	Exclusion Criteria
Dietary or dietary patterns for diabetes; human studies; adults ≥18 years; systematic reviews that included randomised control trials (RCTs) reporting on health outcomes with an appropriate comparator.	Children; adolescent; youth; aged <18 yrs; pregnancy; gestational diabetes; nutritional supplements; isolated foods or nutrients; acute trials; drug trials; animal studies.
Primary outcome of Haemoglobin A1c (HbA1c) with or without other indices of glycaemic control (i.e., fasting blood glucose (FBG), fasting or non-fasting insulin (FBI)).	Nutritional supplements; isolated foods or nutrients; acute trials; drug trials; animal studies; renal or kidney disease; cancer; palliative care.
Secondary findings of weight loss, blood pressure, and blood lipids (total-cholesterol (TC), low density lipoproteins (LDL), high density lipoproteins (HDL) and triglycerides (TG)).	Weight loss surgery or weight loss as single aim. Letters; editorials; commentaries; foreign language studies.
Search terms	
[MeSH terms] Dietary = #1 [Publication date 10 years]	
Advanced–Builder–History: Add #2 Dietary [MeSH terms]	
[All fields] #2 AND [All Fields] “Dietary Approaches to Stop Hypertension” OR [All Fields] DASH OR [All Fields] “Intermittent fasting” OR [All Fields] “Time-restricted feeding” OR [All Fields] “Low-carbohydrate” OR [All Fields] Ketogenic OR [All Fields] “High protein” OR [All Fields] Paleo OR [All Fields] “Low-fat” OR [All Fields] “High carbohydrate” OR [All Fields] “Low-glycaemic” OR [All Fields] “High fibre” OR [All Fields] Prudent OR [All Fields] Mediterranean OR [All Fields] MUFA OR [All Fields] Nordic OR [All Fields] Vegetarian OR Portfolio OR Flexitarian OR Vegan = #3	
[All Fields] diabetes OR [MeSH terms] diabetes = #4	
[Title]–“systematic review” OR [Abstract]–“systematic review” = #5	
[Title] glycaemic OR [Title] glycaemic OR [Title] HbA1c OR glycaemic [Abstract] glycaemic OR [Abstract] HbA1c [Abstract] = #6	
Note: Search terms developed for PubMed Central searches were followed for all databases	
PICO strategy	
Abbreviation-Description	Search components used to review evidence
P-Population	Adults living with diabetes
I-Intervention	Adoption of specific dietary patterns that improve glycaemia
C-Comparator	Control or usual diet for diabetes
O-Outcomes	The primary effects of dietary patterns on glycaemic control and inclusion of secondary findings
(T)-Type of study	Systematic reviews (with or without meta-analysis)

**Table 3:** Systematic reviews comparing dietary patterns (with control diets) included in this review.

Type of Dietary Pattern			
Low-Carbohydrate	Mediterranean	Plant-Based	Low-Glycaemic Index
Systematic reviews with meta-analysis			
Fan, 2016 [51] Goldenberg, 2021 [61] Huntriss, 2018 [45] Jamka, 2020 ^a^ [27] Korsmo-Haugen, 2019 [52] Li, 2021 [62] McArdle, 2019 [53] Meng, 2017 [54] Naude, 2014 [55] Sainsbury, 2018 [56] Snorgaard, 2017 [57] van Zuuren, 2018 [44] Yu, 2020 ^b^ [58] Ajala, 2013 ^e^ [34]	Esposito, 2015 [63] Huo, 2015 [32] Qian, 2016 ^c^ [33] Ajala, 2013 ^e^ [34]	Viguiliouk, 2015 ^d^ [66] Viguiliouk, 2019 ^d^ [67] Yokoyama, 2014 [68]	Ojo, 2019 [39] Zafar, 2019 ^d^ [71] Ajala, 2013 ^e^ [34]
Systematic reviews (without meta-analysis)			
Malaeb, 2019 ^b^ [28] Yamada, 2018 [59] Emadian, 2015 ^e^ [50] Papamichou, 2019 ^e^ [31]	Sleiman, 2015 [65] de Carvalho, 2020 ^e^ [49] Emadian, 2015 ^e^ [50] Papamichou, 2019 ^e^ [31]	Toumpanakis, 2018 [69] Johannesen, 2020 [70] de Carvalho, 2020 ^e^ [49] Emadian, 2015 ^e^ [50] Papamichou, 2019 ^e^ [31]	Emadian, 2015 ^e^ [50] Papamichou, 2019 ^e^ [31]

Note. ^a^ Paleolithic. ^b^ High protein. ^c^ Monounsaturated fatty acid (MUFA). ^d^ includes type 1 diabetes (T1D). ^e^ systematic reviews that compare multiple dietary patterns are incorporated (only counted once and therefore total systematic reviews equal 30).

**Table 4:** Primary outcomes from systematic reviews with meta-analysis.

Type of Dietary Pattern				
	Low-carbohydrate	Mediterranean	Plant-based	Low-glycaemic Index
No. of reviews	*n* = 14 *	*n* = 4 *	*n* = 3	*n* = 3 *
Glycaemic control				
HbA1c	6 Sig favour * MD −0.3% [51], −0.3% [45], −0.4% [54], −0.1% [34], −0.5% [61], −0.4% [62]. 4 Favour, NS MD −0.1% [52], −0.1% [56], −0.04% [57], −0.2% [44]. 4 No difference, NS [27,53,55,58]	3 Sig favour * MD −0.3% [32], −0.5% [63], −0.5% [34] 1 Favour, NS MD −0.1% [33]	3 Sig favour MD −0.2% [66], −0.3% [67], −0.4% [68]	2 Sig favour * MD −0.2% [71], −0.5% [34] 1 Favour, NS MD −0.5% [39]
FBG	5 No difference, NS [27,44,54,58,61] 9 NR * [34,45,51,52,53,55,56,57,62]	2 Sig favour MD −0.7 mmol/L [56] −0.6 mmol/L [57] 2 NR * [34,63]	2 Sig favour MD −0.5 mmol/L [66], MD −0.6 mmol/L [67] 1 No difference [68], NS	2 Sig favour MD −0.2 mmol/L [71], −0.5 mmol/L [39] 1 NR * [34]
FBI	1 No difference, NS [27] 13 NR * [34,44,45,51,52,53,54,55,56,57,58,61,62]	1 Favour, NS MD −0.6 mU/L [32] 1 No difference, NS [33] 2 NR * [34,63]	1 Sig favour WMD −1.5 mU/L [66] 2 NR [67,68]	1 No difference, NS [71] 2 NR * [34,39]

Note. * Includes 1 result from a meta-analysis of multiple dietary patterns. All results are rounded up to one decimal point. All results reported are presented as mean difference MD. Abbreviations: MD = mean difference; *n* = number of reviews; NR = not reported; NS = not significant; Sig = significantly.

**Table 5:** Secondary outcomes from systematic reviews with meta-analysis in four dietary patterns.

Type of Dietary Pattern				
	Low-carbohydrate (LC)	Mediterranean (M)	Plant-based (PB)	Low-glycaemic Index (LGI)
Body weight	*n* = 14 *	*n* = 4 *	*n* = 3	*n* = 3 *
	4 Sig favour * MD −0.7 kg [34], −2.3 kg [51], −3.5 kg [61], −3 kg [62] 1 Favour, NS [52] 8 No difference, NS [27,44,45,53,54,55,56,57] 1 NR [58]	2 Sig favour * MD −1.6 kg [33], −1.8 kg [34] 1 Favour, NS [32] 1 No difference, NS [63]	1 Sig favour MD −2.2 kg [67] 1 No difference, NS [68] 1 NR [66]	2 No difference, NS * [34,71] 1 NR [39]
Blood pressure	*n* = 7	*n* = 2	*n* = 1	*n* = 0
Systolic	1 Sig favour MD −2.7 mm/Hg [45] 6 No difference, NS [44,52,55,56,58,62]	1 Sig favour MD −2.3 mmHg [33] 1 Favours, NS [32]	1 No difference, NS [67]	
Diastolic	7 No difference, NS [44,45,52,55,56,58,62]	1 Favour, NS [32] 1 No difference, NS [33]	1 No difference, NS [67]	
Blood lipids	*n* = 12 *	*n* = 3 *	*n* = 1	*n* = 2 *
TC	1 Sig favour MD 0.2 mmol/L [58] 1 Favour, NS [45] 8 No difference, NS * [34,51,52,54,55,56,61,62] 2 NR [44,57]	1 Sig favour MD −0.1 mmol/L [32] 2 NR * [33,34]	1 NR [67]	1 No difference, NS [71] 1 NR* [34]
LDL	1 Sig favour MD −0.1 mmol/L [58] 11 No difference, NS * [44,45,51,52,54,55,56,57,61,62]	3 No difference, NS * [32,33,34]	1 Sig favour MD −0.12 mmol/L [67]	2 No difference, NS * [34,71]
HDL	5 Sig favour * MD 0.09 mmol/L [51], 0.06 mmol/L [45], 0.07 mmol/L [54], 0.08 mmol/L [34], 0.09 mmol/L [62] 1 Favour, NS [44] 5 No difference, NS [52,55,56,58,61] 1 NR [57]	2 Sig favour * MD 0.06 mmol/L [33], 0.04 mmol/L [34]. 1 Favour, NS [32]	1 No difference, NS [67]	1 Sig favour * MD 0.05 [34] 1 No difference, NS [71]
TG	4 Sig favour MD −0.3 mmol/L [51], −0.2 mmol/L [45], −0.2 mmol/L [58]. −0.2 mmol/L [62] 2 Favour, NS [44,52] 5 No difference, NS * [34,54,55,56,61] 1 NR [57]	3 Sig favour * MD −0.3 mmol/L [32], −0.3 mmol/L [33] −0.2 mmol/L (35).	1 No difference, NS [67]	2 No difference, NS * [34,71]

Note. Results–are rounded up to one decimal point and presented as a mean difference MD (MD or WMD). Abbreviations: Sig = significantly; NS = not significant; NR = not reported; *n* = number of reviews; * = additional result from meta-analysis of multiple dietary patterns; HbA1c = Haemoglobin A1c; FBG = fasting blood glucose; TC = total cholesterol; FBI = fasting blood insulin; LDL = low density lipoprotein; HDL = high density lipoprotein; TG = triglycerides.

**Table 6:** The health benefits of dietary patterns for adults with type 2 diabetes based on this umbrella review.

Health Benefits	HbA1c Reduction	FBG Reduction	Weight Loss	Lowered BP	Reduced TC/TG	Improved HDL
Low-carbohydrate	✓	*	✓	✓	✓	✓
Mediterranean	✓	✓	✓	✓	✓	✓
Plant-based	✓	✓	✓	*	*	*
Low-glycaemic Index	✓	✓	*	*	*	✓

Note. * Insufficient data to draw conclusions. Abbreviations: HbA1c = haemoglobin A1c; FBG = fasting blood glucose; BP = blood pressure; TC = total cholesterol; TG = triglycerides; HDL = high-density lipoprotein. Checkmark denote there was sufficient data consistently demonstrating the dietary pattern significantly improved the outcome.

**Table 7:** Quality and risk of bias from systematic reviews with meta-analysis.

	Low-Carbohydrate		Mediterranean		Plant-Based		Low-Glycaemic Index	
No. of Reviews	*n* = 14 *		*n* = 4 *		*n* = 3		*n* = 3 *	
	Quality	Risk of Bias	Quality	Risk of Bias	Quality	Risk of Bias	Quality	Risk of Bias
	0 High	2 Low	1 High	3 Low	0 High	0 Low	0 High	1 Low
	6 Moderate *	11 Unclear *	3 Moderate *	1 Unclear *	1 Moderate	3 Unclear	2 Moderate *	2 Unclear *
	8 Low	1 High	0 Low	0 High	2 low	0 High	1 Low	0 High

Note. * Additional result from one meta-analysis of multiple dietary patterns (only counted once and therefore total systematic reviews with meta-analysis equal 22).

## Data Availability

Not applicable.

## Supplementary Materials

The following supporting information can be downloaded at: https://www.mdpi.com/article/10.3390/nu15040861/s1. Table S1: Full text records excluded; Table S2: Characteristics of dietary pattern reviews for diabetes; Table S3: Results from reviews on glycaemic control and weight among different dietary patterns: Low carbohydrate versus control diets difference in HbA1c, fasting blood glucose (FBG), fasting blood insulin (FBI) and body weight; Table S4: Results from reviews on glycaemic control and weight among different dietary patterns: Mediterranean versus control diets difference in HbA1c, fasting blood glucose (FBG), fasting blood insulin (FBI) and body weight; Table S5: Results from reviews on glycaemic control and weight among different dietary patterns: Plant-based versus control diets difference in HbA1c, fasting blood glucose (FBG), fasting blood insulin (FBI) and body weight; Table S6: Results from reviews on glycaemic control and weight among different dietary patterns: Low-glycaemic versus control diets difference in HbA1c, fasting blood glucose (FBG), fasting blood insulin (FBI) and body weight; Table S7: Results from reviews on glycaemic control and weight among different dietary patterns: Multi-intervention versus control difference in HbA1c, fasting blood glucose (FBG), fasting blood insulin (FBI) and body weight; Table S8: Results from reviews on cardiovascular markers among different dietary patterns: Dietary interventions versus control diets difference in cardiovascular outcome. Table S9: Conclusions and quality appraisal of dietary patterns for diabetes.
