# Supplementary material for: Determining Dietary Patterns to Recommend for Type 2 Diabetes: An Umbrella Review
Source: Nutrients. 2023 Feb 8;15(4):861. doi: 10.3390/nu15040861 (PMC9958769; doi:10.3390/nu15040861)
Supplement: Supplementary file 1 [file nutrients-15-00861-s001.zip › Table S1_supp.pdf]

Supplementary 1 Full text records excluded

Reasons for exclusion

Records were either interventions about supplements, not including dietary pattern, not reporting glycaemic outcomes, gestational diabetes focus, diet comparator not appropriate, few or no people with diabetes, data not in appropriate format or insufficient, umbrella reviews, focus on development of diabetes, narrative reviews, full text unavailable, foreign language, network meta-analysis, not human study, only prediabetes focus, focus on insulin and type 1 diabetes, included children in studies, protocol only study or scientific statement.

Excluded articles

Anton SD, Hida A, Heekin K, Sowalsky K, Karabetian C, Mutchie H, et al. Effects of Popular Diets without Specific Calorie Targets on Weight Loss Outcomes: Systematic Review of Findings from Clinical Trials. Nutrients. 2017;9(8).

Barreira E, Novo A, Vaz JA, Pereira AM. Dietary program and physical activity impact on biochemical markers in patients with type 2 diabetes: A systematic review. Aten Primaria. 2018;50(10):590-610.

Bogdanet D, Reddin C, Macken E, Griffin TP, Fhelelboom N, Biesty L, et al. Follow-up at 1 year and beyond of women with gestational diabetes treated with insulin and/or oral glucose-lowering agents: a core outcome set using a Delphi survey. Diabetologia. 2019;62(11):2007-16.

Brown TJ, Brainard J, Song F, Wang X, Abdelhamid A, Hooper L, et al. Omega-3, omega-6, and total dietary polyunsaturated fat for prevention and treatment of type 2 diabetes mellitus: systematic review and meta-analysis of randomised controlled trials. Bmj. 2019;366.

Burton T, Alexander J, Planinic P, Basu A. Associations of Plant-Based Foods and Diets with Gestational Diabetes: A Systematic Review of Observational Studies and Clinical Trials. Curr Dev Nutr. 2020;4(Suppl 2):948.

Buyken AE, Goletzke J, Joslowski G, Felbick A, Cheng G, Herder C, et al. Association between carbohydrate quality and inflammatory markers: systematic review of observational and interventional studies. The American journal of clinical nutrition. 2014;99(4):813-33.

Campbell GJ, Senior AM, Bell-Anderson KS. Metabolic Effects of High Glycaemic Index Diets: A Systematic Review and Meta-Analysis of Feeding Studies in Mice and Rats. Nutrients. 2017;9(7).

Carter P, Achana F, Troughton J, Gray LJ, Khunti K, Davies MJ. A Mediterranean diet improves HbA1c but not fasting blood glucose compared to alternative dietary strategies: a network meta-analysis. J Hum Nutr Diet. 2014;27(3):280-97.

Chaudhry ZW, Doshi RS, Mehta AK, Jacobs DK, Vakil RM, Lee CJ, et al. A systematic review of commercial weight loss programmes' effect on glycemic outcomes among overweight and obese adults with and without type 2 diabetes mellitus. Obes Rev. 2016;17(8):758-69.

Chiavaroli L, Kendall CWC, Braunstein CR, Mejia SB, Leiter LA, Jenkins DJA, et al. Effect of pasta in the context of low-glycaemic index dietary patterns on body weight and markers of adiposity: a systematic review and meta-analysis of randomised controlled trials in adults. BMJ Open. 2018;8(3):13.

Chiavaroli L, Lee D, Ahmed A, Cheung A, Khan TA, Blanco S, et al. Effect of low glycaemic index or load dietary patterns on glycaemic control and cardiometabolic risk factors in diabetes: systematic review and meta-analysis of randomised controlled trials. BMJ. 2021;374:n1651.

Chiavaroli L, Viguiliouk E, Nishi SK, Blanco Mejia S, Rahelić D, Kahleová H, et al. DASH Dietary Pattern and Cardiometabolic Outcomes: An Umbrella Review of Systematic Reviews and Meta-Analyses. Nutrients. 2019;11(2).

Cho Y, Hong N, Kim K, Cho S, Lee M, Lee Y, et al. The Effectiveness of Intermittent Fasting to Reduce Body Mass Index and Glucose Metabolism: A Systematic Review and Meta-Analysis. J Clin Med. 2019;8(10).

Churuangsuk C, Hall J, Reynolds A, Griffin SJ, Combet E, Lean MEJ. Diets for weight management in adults with type 2 diabetes: an umbrella review of published meta-analyses and systematic review of trials of diets for diabetes remission. Diabetologia. 2022;65(1):14-36.

Clar C, Al-Khudairy L, Loveman E, Kelly SAM, Hartley L, Flowers N, et al. Low glycaemic index diets for the prevention of cardiovascular disease. Cochrane Database of Systematic Reviews. 2017(7).

Dening J, Islam SMS, George E, Maddison R. Web-Based Interventions for Dietary Behavior in Adults With Type 2 Diabetes: Systematic Review of Randomized Controlled Trials. J Med Internet Res. 2020;22(8):13.

Dinu M, Pagliai G, Angelino D, Rosi A, Dall'Asta M, Bresciani L, et al. Effects of Popular Diets on Anthropometric and Cardiometabolic Parameters: An Umbrella Review of Meta-Analyses of Randomized Controlled Trials. Adv Nutr. 2020;11(4):815-33.

Fan J, Song Y, Wang Y, Hui R, Zhang W. Dietary Glycemic Index, Glycemic Load, and Risk of Coronary Heart Disease, Stroke, and Stroke Mortality: A Systematic Review with Meta-Analysis. PLoS One. 2012;7(12).

Franz MJ, Boucher JL, Rutten-Ramos S, VanWormer JJ. Lifestyle Weight-Loss Intervention Outcomes in Overweight and Obese Adults with Type 2 Diabetes: A Systematic Review and Meta-Analysis of Randomized Clinical Trials. J Acad Nutr Diet. 2015;115(9):1447-63.

Gilinsky A, Kirk A, Hughes A, Lindsay R. Lifestyle interventions for type 2 diabetes prevention in women with prior gestational diabetes: A systematic review and meta-analysis of behavioural, anthropometric and metabolic outcomes☆. Prev Med Rep. 2015;2:448-61.

Goff LM, Cowland DE, Hooper L, Frost GS. Low glycaemic index diets and blood lipids: A systematic review and meta-analysis of randomised controlled trials. Nutr Metab Carbiovasc Dis. 2013;23(1):1-10.

Greenwood DC, Threapleton DE, Evans CEL, Cleghorn CL, Nykjaer C, Woodhead C, et al. Glycemic index, glycemic load, carbohydrates, and type 2 diabetes: systematic review and dose-response meta-analysis of prospective studies. Diabetes Care. 2013;36(12):4166-71.

Ha V, Bonner AJ, Jadoo JK, Beyene J, Anand SS, de Souza RJ. The effects of various diets on glycemic outcomes during pregnancy: A systematic review and network meta-analysis. PLoS One. 2017;12(8):17.

Hallberg SJ, Gershuni VM, Hazbun TL, Athinarayanan SJ. Reversing type 2 diabetes: a narrative review of the evidence. Nutrients. 2019;11(4):766.

Hernández Alcantara G, Jiménez Cruz A, Bacardí Gascón M. Effect of low carbohydrate diets on weight loss and glycosilate hemoglobin in people with type 2 diabetes: Sytematic Review. Nutr Hosp. 2015;32(5):1960-6.

Huang YS, Zheng QY, Yang HS, Fu XW, Zhang XQ, Xia CH, et al. Efficacy of Intermittent or Continuous Very Low-Energy Diets in Overweight and Obese Individuals with Type 2 Diabetes Mellitus: A Systematic Review and Meta-Analyses. J Diabetes Res. 2020;2020:21.

Imamura F, Micha R, Wu JHY, de Oliveira Otto MC, Otite FO, Abioye AI, et al. Effects of Saturated Fat, Polyunsaturated Fat, Monounsaturated Fat, and Carbohydrate on Glucose-Insulin Homeostasis: A Systematic Review and Meta-analysis of Randomised Controlled Feeding Trials. PLoS Med. 2016;13(7).

Kirkpatrick CF, Bolick JP, Kris-Etherton PM, Sikand G, Aspry KE, Soffer DE, et al. Review of current evidence and clinical recommendations on the effects of low-carbohydrate and very-low-carbohydrate (including ketogenic) diets for the management of body weight and other cardiometabolic risk factors: A scientific statement from the National Lipid Association Nutrition and Lifestyle Task Force. Journal of clinical lipidology. 2019;13(5):689-711.e1.

Koloverou E, Esposito K, Giugliano D, Panagiotakos D. The effect of Mediterranean diet on the development of type 2 diabetes mellitus: A meta-analysis of 10 prospective studies and 136,846 participants. Metab-Clin Exp. 2014;63(7):903-11.

Louie JCY, Brand-Miller JC, Markovic TP, Ross GP, Moses RG. Glycemic Index and Pregnancy: A Systematic Literature Review. J Nutr Metab. 2010;2010.

Luna López V, López Medina JA, Vázquez Gutiérrez M, Fernández Soto ML. [Carbohydrate: current role in diabetes mellitus and metabolic disease]. Nutr Hosp. 2014;30(5):1020-31.

Massara P, Viguiliouk E, Glenn A, Khan T, Chiavaroli L, Mejia SB, et al. Nordic Dietary Pattern and Cardiometabolic Outcomes: A Systematic Review and Meta-Analysis of Prospective Cohort Studies and Randomized Controlled Trials. Curr Dev Nutr. 2020;4(Suppl 2):546.

Mathews MJ, Liebenberg L, Mathews EH. How do high glycemic load diets influence coronary heart disease? Nutr Metab (Lond). 2015;12.

Maula A, Kai J, Woolley AK, Weng S, Dhalwani N, Griffiths FE, et al. Educational weight loss interventions in obese and overweight adults with type 2 diabetes: a systematic review and meta-analysis of randomized controlled trials. Diabetic Med. 2020;37(4):623-35.

Maula A, Kendrick D, Kai J, Griffiths F. Evidence generated from a realist synthesis of trials on educational weight loss interventions in type 2 diabetes mellitus. Diabetic Med.16.

Muilwijk M, Nicolaou M, Qureshi SA, Celis-Morales C, Gill JMR, Sheikh A, et al. Dietary and physical activity recommendations to prevent type 2 diabetes in South Asian adults: A systematic review. PLoS One. 2018;13(7).

Neuenschwander M, Hoffmann G, Schwingshackl L, Schlesinger S. Impact of different dietary approaches on blood lipid control in patients with type 2 diabetes mellitus: a systematic review and network meta-analysis. Eur J Epidemiol. 2019;34(9):837-52.

Ni C, Jia Q, Ding G, Wu X, Yang M. Low-Glycemic Index Diets as an Intervention in Metabolic Diseases: A Systematic Review and Meta-Analysis. Nutrients. 2022;14(2).

Noto H, Goto A, Tsujimoto T, Noda M. Low-Carbohydrate Diets and All-Cause Mortality: A Systematic Review and Meta-Analysis of Observational Studies. PLoS One. 2013;8(1):10.

Ojo O, Ojo OO, Wang XH, Adegboye ARA. The Effects of a Low GI Diet on Cardiometabolic and Inflammatory Parameters in Patients with Type 2 and Gestational Diabetes: A Systematic Review and Meta-Analysis of Randomised Controlled Trials. Nutrients. 2019;11(7).

Palma-Duran SA, Vlassopoulos A, Lean M, Govan L, Combet E. Nutritional intervention and impact of polyphenol on glycohemoglobin (HbA1c) in non-diabetic and type 2 diabetic subjects: Systematic review and meta-analysis. Crit Rev Food Sci Nutr. 2017;57(5):975-86.

Paterson MA, King BR, Smart CEM, Smith T, Rafferty J, Lopez PE. Impact of dietary protein on postprandial glycaemic control and insulin requirements in Type 1 diabetes: a systematic review. Diabetic Med. 2019;36(12):1585-99.

Philippou E, Constantinou M. The influence of glycemic index on cognitive functioning: a systematic review of the evidence. Advances in nutrition (Bethesda, Md). 2014;5(2):119-30.

Pinto CL, Botelho PB, Pimentel GD, Campos-Ferraz PL, Mota JF. Creatine supplementation and glycemic control: a systematic review. Amino acids. 2016;48(9):2103-29.

Poolsup N, Suksomboon N, Aung N. Effect of phosphodiesterase-5 inhibitors on glycemic control in person with type 2 diabetes mellitus: A systematic review and meta-analysis. J Clin Transl Endocrinol. 2016;6:50-5.

Pundir J, Charles D, Sabatini L, Hiam D, Jitpiriyaroj S, Teede H, et al. Overview of systematic reviews of non-pharmacological interventions in women with polycystic ovary syndrome. Human reproduction update. 2019;25(2):243-56.

Ranasinghe P, Jayawardena R, Pigera ASAD, Katulanda P, Constantine GR, Galappaththy P. Zinc supplementation in pre-diabetes: study protocol for a randomized controlled trial. Trials. 2013;14:52.

Rao M, Gao C, Xu L, Jiang L, Zhu J, Chen G, et al. Effect of Inulin-Type Carbohydrates on Insulin Resistance in Patients with Type 2 Diabetes and Obesity: A Systematic Review and Meta-Analysis. J Diabetes Res. 2019;2019:5101423.

Reynolds A, Mann J, Cummings J, Winter N, Mete E, Te Morenga L. Carbohydrate quality and human health: a series of systematic reviews and meta-analyses. Lancet (London, England). 2019;393(10170):434-45.

Reynolds AN, Akerman AP, Mann J. Dietary fibre and whole grains in diabetes management: Systematic review and meta-analyses. PLos Med. 2020;17(3):22.

Ribeiro PVM, Silva A, Almeida AP, Hermsdorff HH, Alfenas RC. Effect of chronic consumption of pistachios (Pistacia vera L.) on glucose metabolism in pre-diabetics and type 2 diabetics: A systematic review. Crit Rev Food Sci Nutr. 2019;59(7):1115-23.

Santesso N, Akl EA, Bianchi M, Mente A, Mustafa R, Heels-Ansdell D, et al. Effects of higher- versus lower-protein diets on health outcomes: a systematic review and meta-analysis. Eur J Clin Nutr. 2012;66(7):780-8.

Schwingshackl L, Chaimani A, Hoffmann G, Schwedhelm C, Boeing H. Impact of different dietary approaches on glycemic control and cardiovascular risk factors in patients with type 2 diabetes: a protocol for a systematic review and network meta-analysis. Syst Rev. 2017;6:7.

Schwingshackl L, Chaimani A, Hoffmann G, Schwedhelm C, Boeing H. A network meta-analysis on the comparative efficacy of different dietary approaches on glycaemic control in patients with type 2 diabetes mellitus. Eur J Epidemiol. 2018;33(2):157-70.

Schwingshackl L, Hoffmann G. Long-term effects of low-fat diets either low or high in protein on cardiovascular and metabolic risk factors: a systematic review and meta-analysis. Nutr J. 2013;12:48.

Shahdadian F, Mohammadi H, Rouhani MH. Effect of Vitamin K Supplementation on Glycemic Control: A Systematic Review and Meta-Analysis of Clinical Trials. Hormone and metabolic research = Hormon- und Stoffwechselforschung = Hormones et metabolisme. 2018;50(3):227-35.

Shirani F, Salehi-Abargouei A, Azadbakht L. Effects of Dietary Approaches to Stop Hypertension (DASH) diet on some risk for developing type 2 diabetes: A systematic review and meta-analysis on controlled clinical trials. Nutrition. 2013;29(7-8):939-47.

Silva FM, Kramer CK, de Almeida JC, Steemburgo T, Gross JL, Azevedo MJ. Fiber intake and glycemic control in patients with type 2 diabetes mellitus: a systematic review with meta-analysis of randomized controlled trials. Nutr Rev. 2013;71(12):790-801.

Simental-Mendía LE, Sahebkar A, Rodríguez-Morán M, Guerrero-Romero F. A systematic review and meta-analysis of randomized controlled trials on the effects of magnesium supplementation on insulin sensitivity and glucose control. Pharmacological research. 2016;111:272-82.

Snelson M, Jong J, Manolas D, Kok S, Louise A, Stern R, et al. Metabolic Effects of Resistant Starch Type 2: A Systematic Literature Review and Meta-Analysis of Randomized Controlled Trials. Nutrients. 2019;11(8).

Sudchada P, Saokaew S, Sridetch S, Incampa S, Jaiyen S, Khaithong W. Effect of folic acid supplementation on plasma total homocysteine levels and glycemic control in patients with type 2 diabetes: a systematic review and meta-analysis. Diabetes Res Clin Pract. 2012;98(1):151-8.

Suksomboon N, Poolsup N, Juanak N. Effects of coenzyme Q10 supplementation on metabolic profile in diabetes: a systematic review and meta-analysis. Journal of clinical pharmacy and therapeutics. 2015;40(4):413-8.

Suksomboon N, Poolsup N, Sinprasert S. Effects of vitamin E supplementation on glycaemic control in type 2 diabetes: systematic review of randomized controlled trials. Journal of clinical pharmacy and therapeutics. 2011;36(1):53-63.

Suksomboon N, Poolsup N, Yuwanakorn A. Systematic review and meta-analysis of the efficacy and safety of chromium supplementation in diabetes. Journal of clinical pharmacy and therapeutics. 2014;39(3):292-306.

Tabatabaei-Malazy O, Nikfar S, Larijani B, Abdollahi M. Influence of ascorbic acid supplementation on type 2 diabetes mellitus in observational and randomized controlled trials; a systematic review with meta-analysis. Journal of pharmacy & pharmaceutical sciences : a publication of the Canadian Society for Pharmaceutical Sciences, Societe canadienne des sciences pharmaceutiques. 2014;17(4):554-82.

Thomaz de Lima H, Lopes Rosado E, Ribeiro Neves PA, Corrêa Monteiro Machado R, Mello de Oliveira L, Saunders C. Systematic review; Nutritional therapy in gestational diabetes mellitus. Nutr Hosp. 2013;28(6):1806-14.

Tobias DK, Chen M, Manson JE, Ludwig DS, Willett W, Hu FB. Effect of low-fat diet interventions versus other diet interventions on long-term weight change in adults: a systematic review and meta-analysis. Lancet Diabetes Endocrinol. 2015;3(12):968-79.

Turton J, Brinkworth GD, Field R, Parker H, Rooney K. An evidence-based approach to developing low-carbohydrate diets for type 2 diabetes management: A systematic review of interventions and methods. Diabetes Obes Metab. 2019;21(11):2513-25.

Turton JL, Raab R, Rooney KB. Low-carbohydrate diets for type 1 diabetes mellitus: A systematic review. PLoS One. 2018;13(3).

Valenzuela Mencía J, Fernández Castillo R, Martos Cabrera MB, Gómez-Urquiza JL, Albendín García L, Cañadas de la Fuente GA. Diets low in carbohydrates for type 2 diabetics. Systematic review. Nutr Hosp. 2017;34(1):224-34.

van Baak MA, Mariman ECM. Dietary Strategies for Weight Loss Maintenance. Nutrients. 2019;11(8).

Viana LV, Gross JL, Azevedo MJ. Dietary Intervention in Patients With Gestational Diabetes Mellitus: A Systematic Review and Meta-analysis of Randomized Clinical Trials on Maternal and Newborn Outcomes. Diabetes Care. 2014;37(12):3345-55.

Vitale R, Kim Y. The Effects of Intermittent Fasting on Glycemic Control and Body Composition in Adults with Obesity and Type 2 Diabetes: A Systematic Review. Metab Syndr Relat Disord. 2020:12.

Wan CS, Nankervis A, Teede H, Aroni R. Dietary intervention strategies for ethnic Chinese women with gestational diabetes mellitus: A systematic review and meta-analysis. Nutrition & dietetics: the journal of the Dietitians Association of Australia. 2019;76(2):211-32.

Wang L, Yang H, Huang H, Zhang C, Zuo H-X, Xu P, et al. Inulin-type fructans supplementation improves glycemic control for the prediabetes and type 2 diabetes populations: results from a GRADE-assessed systematic review and dose-response meta-analysis of 33 randomized controlled trials. Journal of translational medicine. 2019;17(1):410.

Wang S, Wang R, Zhang Y, Zhang X, Cai B, Lu Y, et al. Therapies for diabetic gastroparesis: A protocol for a systematic review and network meta-analysis. Medicine (Baltimore). 2020;99(21).

Wang X, Tian J, Jiang J, Li L, Ying X, Tian H, et al. Effects of green tea or green tea extract on insulin sensitivity and glycaemic control in populations at risk of type 2 diabetes mellitus: a systematic review and meta-analysis of randomised controlled trials. Journal of human nutrition and dietetics : the official journal of the British Dietetic Association. 2014;27(5):501-12.

Wang X, Wu W, Zheng W, Fang X, Chen L, Rink L, et al. Zinc supplementation improves glycemic control for diabetes prevention and management: a systematic review and meta-analysis of randomized controlled trials. The American journal of clinical nutrition. 2019;110(1):76-90.

Welton S, Minty R, O'Driscoll T, Willms H, Poirier D, Madden S, et al. Intermittent fasting and weight loss: Systematic review. Can Fam Physician. 2020;66(2):117-25.

Wheeler ML, Dunbar SA, Jaacks LM, Karmally W, Mayer-Davis EJ, Wylie-Rosett J, et al. Macronutrients, Food Groups, and Eating Patterns in the Management of Diabetes A systematic review of the literature, 2010. Diabetes Care. 2012;35(2):434-45.

Wycherley TP, Moran LJ, Clifton PM, Noakes M, Brinkworth GD. Effects of energy-restricted high-protein, low-fat compared with standard-protein, low-fat diets: a meta-analysis of randomized controlled trials. Am J Clin Nutr. 2012;96(6):1281-98.

Yuan X, Wang J, Yang S, Gao M, Cao L, Li X, et al. Effect of the ketogenic diet on glycemic control, insulin resistance, and lipid metabolism in patients with T2DM: a systematic review and meta-analysis. Nutr Diabetes. 2020;10(1):38.

Zaki HA, Iftikhar H, Bashir K, Gad H, Samir Fahmy A, Elmoheen A. A Comparative Study Evaluating the Effectiveness Between Ketogenic and Low-Carbohydrate Diets on Glycemic and Weight Control in Patients With Type 2 Diabetes Mellitus: A Systematic Review and Meta-Analysis. Cureus. 2022;14(5):e25528.

Zhang X, Shi Z, Liu Q, Quan H, Cheng X. Effects of coenzyme Q10 intervention on diabetic kidney disease: A systematic review and meta-analysis. Medicine (Baltimore). 2019;98(24).

Zhang Y, Liu W, Liu D, Zhao T, Tian H. Efficacy of Aloe Vera Supplementation on Prediabetes and Early Non-Treated Diabetic Patients: A Systematic Review and Meta-Analysis of Randomized Controlled Trials. Nutrients. 2016;8(7).

Zhao JV, Schooling CM, Zhao JX. The effects of folate supplementation on glucose metabolism and risk of type 2 diabetes: a systematic review and meta-analysis of randomized controlled trials. Annals of epidemiology. 2018;28(4):249-57.e1.
