# Supplementary material for: Determining Dietary Patterns to Recommend for Type 2 Diabetes: An Umbrella Review
Source: Nutrients. 2023 Feb 8;15(4):861. doi: 10.3390/nu15040861 (PMC9958769; doi:10.3390/nu15040861)
Supplement: Supplementary file 1 [file nutrients-15-00861-s001.zip › Table S2_supp.pdf]

Supplementary Table S2 Characteristics of dietary pattern reviews for diabetes

**Low carbohydrate**						
**Author of study**	**Countries**	**No. of studies**	**Participants (n= number) Age (yrs.)**	**Intervention/ objective (CHO % energy/day)**	**Comparison/ control (CHO % energy/ day)**	**Duration**
			**Systematic review with meta-analysis A**			
Fan, 2016	4 USA, 2 Israel, 1 Sweden, 1 Italy, 1 UK, 1 Japan	10 RCTs	T2D (n= 997) Age ≥ 18 Δ	Effect of LCD on glycemic control, lipids, and wt loss. (20-60g, 30-130g)	Control LF or calorie energy restricted or macro-nutrient (CHO 50-60%, Fat <30% Protein <20%)	12 to 224 weeks
Goldenberg, 2021 #	Multiple NR	23 Trials	T2D (n=1357) Age 47-67	Efficacy, safety, certainty of LC diet outcomes. (<26% or <130g, <10% or <50g)	Control in 18 of 23 used LF diets	25 to 52 weeks
Huntriss, 2018	Multiple NR	18 RCTs (7 in MA)	T2D (n= 2204) Age ≥ 18 Δ	Effect of LC diet (various) on glycemic control. (20-52%, or <20-130g)	Usual care diets, (>48%)	12 to 224 weeks
Jamka, 2020 a	2 Sweden, 1 Netherlands, 1 USA	4 RCTs (3 in MA)	GMD+ (n= 98) Age: 52-66 (SD 6-13) □	Paleolithic diet effect on glucose and insulin homeostasis. (32-52%)	Various healthy diets (M diet, diabetes diet or Dutch Health Council diet) HE diets (42-52%)	2 to 12 weeks
Korsmo-Haugen, 2019	2 USA (4 USA, 5 Australia, 3 Israel, 3 Sweden, 2 Canada, 1 New Zealand, 1 Austria, 1 Japan, 1 UK)	23 RCTs (16 in MA)	T2D (n= 2178) Age: NR	Effects of LC diets on body wt, lipid profile, BP & glycaemic control. (5-40% or 39g)	Higher CHO diets (40-60% to 52g)	14 to 112 weeks
Li, 2021	3 USA, 2 Sweden, 2 Japan, 1 China, 1 UK, Spain, 1 Australia, 1 Italy	12 RCTs	T2D (n=761) Age: 53-65 □	Assess difference between LC diets and LF diets. LC diet (20-50g, <20%, <130g)	LF diet (<25-30%) or HC (50-60%)	13 to 104 weeks

McArdle, 2019	6 Australia, 4 USA, 3 UK, 2 Israel, 2 Sweden, 2 Japan, 2 New Zealand, 1 Israel, 1 Canada, 1 Italy, 1 Malaysia	25 RCTs	T2D (n= 2132) Age: 52-64 □	CHO restriction compared for HbA1c, and CHO consumed. VLCD: 20-50g, LCD: 70-130g MCD: 128-232g	LF, low calorie, M diet, or LF (AHA), ADA, LF, HC, LGI, JDA, low calorie, DM diet, LP. (CHO: 50-60%)	12 to 208 weeks
Meng, 2017	4 USA, 1 Australia, 1 UK, 1 Israel, 1 Sweden, 1 Japan	9 RCTs	T2D (n= 734) Age: NR	Efficacy of LC diets (5-20% to 20-130g)	Normal or HC diets (50-60% to unclear)	12 to 112 weeks
Naude, 2014 #	5 USA, 4 Australia, 2 Spain, 1 UK, 1 Germany, 1 Norway, (1 Sweden, 3 Australia, 1 New Zealand)	19 studies (5 RCTs T2D)	O/ wt. or obese **#**T2D (n= 720) Age: 39-73 MR	Wt loss diets as LC more effective; to prevent CVD. T2D subgroups. Included HFD and HPD (<45%)	Isoenergetic balanced wt loss diets (CHO >65%)	12 to 112 weeks
Sainsbury, 2018	7 USA, 6 Australia, 2 UK, 2 Canada, 1 Israel, 1 Sweden, 1 Japan, 1 Czech Republic, 1 New Zealand, 1 Austria, 1 Japan	25 RCTs (12 in MA)	T2D (T1D) (n= 2412) Age: 52-63 □ (T1D 37 .9 Mean)	CHO restricted diets effects on glycaemic control. (<10%, <26%, MCD 26-45%, ≤45%)	Comparison with HC diet (CHO ≥45%)	10 to 112 weeks
Snorgaard, 2017	3 USA, 2 Australia, 1 Israel, 1 Sweden, 1 Canada 1 New Zealand, 1 Japan,	10 RCTs (8 in MA)	T2D (n= 1376) Age: 54-62 □	Diets with low to moderate CHO, LCD (14-42%, E% <45%)	Compared to diets with HC. (CHO 48-55%)	12 to 112 weeks
van Zuuren, 2018	12 USA, 9 UK, 2 Australia, 2 Israel, 2 Spain, 2 Japan, 1 Sweden, 1 Italy, 1 Mexico, 1 Canada (2 USA, 1 Sweden CCTs)	36 studies (33 RCTs, 3 CCTs) 14 in MA	T2D (n= 2161) Age: 32-65 MR	LC diets reduced markers of metabolic syndrome; diet (10-40% to 20-130g, ≤40% of energy).	Compared to LF diets (45-70%) fat ≤30%	≥4 weeks

Yu, 2020 b	Multiple NR	13 RCTs	T2D (n= 1138) Age: 54- 63.7 □	Effects of a HP diets (Protein >25%, 25-33%, CHO: 14 -45%) on glycemic control, HOMA IR & BP.	HC diets (CHO 45-60%, Protein: 15-21%)	>12 weeks
**Systematic reviews with no meta-analysis B**						
Malaeb, 2019 b	Multiple NR	21 studies (7 RCTs)	T2D (n= 906) Age: NR	HP diets (Protein 30%, 17-62%) to improve glycemic outcomes. (HP ≥typical US diet (>16% of TE).	Control diet as protein. Some uncontrolled interventions. (P: 15-20%)	2 to 42 weeks
Yamada, 2018	3 Japan	3 RCTs	T2D (n= 105) Age: 54.3-63.3 MR	Effect of energy restricted & CHO restricted diets (70-180g or ER 1800 kcal)	ER or HC diets 2 studies (1 E restricted, 1 CHO rich)	1 to 28 weeks
**Mediterranean**						
**Author of study**	**Country**	**No. of studies**	**Participants (n= number) Age (yrs.)**	**Intervention/ objective (CHO % energy/day)**	**Comparison/ control (CHO % energy/ day)**	**Duration**
**Systematic review with meta-analysis A**						
Esposito, 2015	Multiple NR	8 MA (4 T2D), 5 RCTs (3 T2D)	T2D adults with or at risk (n= 2840) Age: NR	Diets describing themselves as M diet to improve glycaemic control.	Control diets, mainly low-fat diets.	At least 26 weeks
Huo, 2015	3 USA, 2 Israel, 1 Australia, 1 Spain, 1 Italy, 1 Greece	9 RCTs	T2D (n= 1178) Age: 26-75	Mediterranean-style diets, rich in fibre, vegetables, legumes, fruit, fish, MUFA, and limited red, processed meat and animal fats. [CHO: 50-60%, Protein: 15-20%, Fat: 10-40%]	High CHO diets, LFD, regular diets, usual care, or American Diabetes Association. [CHO: 50-60%, Protein: 15-20%, Fat: 10-40%]	At least 4 weeks

Qian, 2016 c	Multiple NR	28 RCTs (24 MUFA vs. CHO)	T2D (n= 1,460) Age: 45-73.9 □	Diets high in MUFA improve T2D metabolic risk factors. [CHO: 10-60%, Protein: 13-60%, Fat: 30-69 (17-49 MUFA)]	High CHO diets (or MUFA vs. PUFA diets). Some trials more than two arms [CHO:41-64: Protein 12-23, Fat: 10-49 (1-19% MUFA)]	2 to 52 weeks
			**Systematic review with no meta-analysis B**			
Sleiman, 2015	Multiple NR	7 RCTs (4 RCTs, 3 T2D subgroup)	T2D or subgroup (n= 1,428) Age: NR	MDiet favorable for glycaemic control. Rich in fruit and veg, bread, cereals, olive oil as fat, low-mod fish, poultry, alcohol, and little red meat.	Compared with LFD or usual dietary habits.	12 to 224 weeks

Plant-based diets

Study author	Country	No. of studies	Participants (n= number) Age (yrs.)	Intervention/ objective (CHO % energy/day)	Comparison/ control (CHO % energy/ day)	Duration
			**Systematic review with meta-analyses A**			
Viguiliouk, 2015 d	5 Iran, 3 USA, 1 Canada, 1 Greece 1 Denmark, 1 Germany	13 RCTs (9 in MA)	T2D & T1D (n= 280) Age: 30-68 □	Effect of plant protein on glycemic control. 40-70% energy from CHO (2.5-64g)	Includes animal protein, match for energy (isocaloric)	≥3 weeks
Viguiliouk, 2019 d	5 USA, 1 Greece, 1 Brazil, 1 Czech Republic, 1 Korea	9 RCTs	T2D & T1D (n= 664) Age: 32-61 □	Effect of vegetarian diets (vegan, lacto-ovo) on HbA1c and risk factors. (49-78: 12-17: 11-34)	Non-vegetarian dietary patterns (47-65: 16-22: 19-37)	≥3 weeks
Yokoyama, 2014	2 USA, 1 Czech Republic, 1 Brazil (2 USA CTs)	6 studies (4 RCTs, 2 CTs)	T2D (n= NR) Age: 44.4-59 □	Vegetarian diets for glycemic control. (5 LF vegan studies 1 lacto-ovo).	Comparator diets include Omnivorous, diabetic diet, ADA diet or LFD.	4 - 74 weeks
			**Systematic reviews with no meta-analysis B**			

Toumpanakis, 2018	4 USA, 2 Czech Republic, 1 New Zealand, 1 Italy, 1 South Korea (2 USA CTs)	13 studies (11 RCTs, 2 CTs) 8 T2D	T2D (n= 433) Age: 44-66 □	Plant-based diet interventions targeting well-being for diabetes. (LF vegan, LF Plant-based, Vegan, Plant-based).	Omnivorous, LF, KDA, ADA, Italian diabetology doctors, European DM study.	3 - 74 weeks
Johannesen, 2020	Multiple NR	9 studies (3 RCTs, 6 RTs) 3 including	T2D, o/ wt. or obese/ CVD (n= 250) Age: 50-61 MR	Plant-based diet effect on glucose metabolism. (LF plant-based, LF vegan, Low-calorie lacto-ovo vegetarian, Lacto-ovo vegetarian, Vegan, LF-LGI vegan)	Conventional omnivorous diet (or portion controlled), Low calorie-M diet or diet from KDA, ADA or AHA.	4 - 22 weeks
**Low glycaemic index**						
**Author of study**	**Country**	**No. of studies**	**Participants (n= number) Age (yrs.)**	**Intervention/ objective (CHO % energy/day)**	**Comparison/ control (CHO % energy/ day)**	**Duration**
			**Systematic review with meta-analysis**			
Ojo, 2018	Multiple NR	9 RCTs (6 with MA)	T2D (n= 705) Age: 42.4-63 □	Effect of LGI diets with T2D. (GI ≤40 and GI ≤55 for low-GI diet)	Higher-GI diet (GI ≥ 70 for high-GI diet) and/or control diet.	2 weeks to 103 weeks
Zafar, 2019 #	Multiple NR	54 RCTs (23 T2D#)	T2D (n= #1424) Age: NR	Assess effects of LGI on glycaemic control.	High GI diets	1 to 52 weeks
**Multiple intervention diets**						
**Author of study**	**Country**	**No. of studies**	**Participants (n= number) Age (yrs.)**	**Intervention/ objective (CHO % energy/day)**	**Comparison/ control (CHO % energy/ day)**	**Duration**
			**Systematic review with meta-analysis A**			
Ajala, 2013	Multiple NR	20 RCTs (16 in MA)	T2D (n= 3073: LC= 844, M= 1397, PB= 173, LGI= 2129) Age: ≥ 18 Δ	Effect of various diets on glycemic control, lipids & wt. loss (LC, vegetarian, vegan, LGI, high-fiber, M diet, HP diets).	LF, High-GI, LP or ADA diet or European Association for Study of Diabetes Guidelines.	≥26 weeks
			**Systematic review with no meta-analysis B**			

de Carvalho, 2020	1 USA, 1 Italy, 1 Iran, 1 Czech Republic, 1 Canada, 1 Korea	6 studies (4 RCTs) 1 cross-over 1 cohort	T2D (n= 690) Age: 30-82	Different dietary patterns for glycaemic control. (LF vegan diet (%CHO 75: P 15: F 10) M diet (F: <30%, CHO <50%) veg diet (%CHO 60: P 15: F: 25), DASH diet).	Conventional diet (%CHO 60-70: P 15-20:SFA<7), LF diet (<30% SFA<7%), diabetic diet (%CHO 50: P 20: F 30 SFA>7%).	8 to 20 weeks
Emadian, 2015	Multiple NR	11 RCTs	T2D, o/ wt. or obese (n= 1266) Age: NR	Effect on glycaemic control with LF vegan, LGI, HP, standard protein, LC, low GL, LC, TM, HC-fibre & modified lipid diets.	Various control diets. Studies had at least two arms. LF, ADA diet, HC diets, standard protein.	Minimum 26 weeks
Papamichou, 2019	Multiple NR	20 RCTs	T2D (n= 2223) Age: >18 Δ	Effect of LC, macrobiotic, vegan, vegetarian, M diet, on glycemic control.	LF diet	≥28 weeks

Note. One systematic review with meta-analyses and three systematic reviews examined multiple intervention diets for people with type 2 diabetes including those overweight or obese. a = Paleolithic diets. b = High protein diets. c = Monounsaturated fatty acid (MUFA). d = includes type 1 diabetes (T1D).# = subgroup data. Δ = No upper limit for age reported. □ = Age as range of means. Abbreviations: ADA = American Diabetes Association; AHA = American Heart Association; Ax = assessment; CCTs = controlled clinical trials; CHO = carbohydrate; CI, = confidence interval; CVD = cardiovascular disease; DA = Diabetes Association; DASH = dietary approaches to stop hypertension; FBG = fasting blood glucose; GDM = gestational diabetes mellitus; LGI = Low-glycaemic Index; GL = glycaemic load; GMD = glucose metabolism disorders; HbA1c = haemoglobin A1c ; HC, high carbohydrate; JDA = Japanese Diabetes Association; KDA = Korean Diabetes Association ; LC = low carbohydrate; LF = low fat; LP = low protein; MA = meta-analysis ; M diet = Mediterranean diet; MS = Mean sample; MUFA = monounsaturated fatty acid ; Mx = management ; NR = not reported ; NS = no significant differences ; PUFA = polyunsaturated fatty acid; PWD = people with diabetes; RCT = randomized control trial SR = systematic review; T1D = type 1 diabetes; T2D = type 2 diabetes; TE = total energy; TM = traditional Mediterranean; UK = United Kingdom; Wt = weight.
