# Supplementary material for: Determining Dietary Patterns to Recommend for Type 2 Diabetes: An Umbrella Review
Source: Nutrients. 2023 Feb 8;15(4):861. doi: 10.3390/nu15040861 (PMC9958769; doi:10.3390/nu15040861)
Supplement: Supplementary file 1 [file nutrients-15-00861-s001.zip › Table S3_supp.pdf]

Supplementary Table S3 Results from reviews on glycaemic control and weight among different dietary patterns: Low carbohydrate versus control diets difference in HbA1c, fasting blood glucose (FBG), fasting blood insulin (FBI) and body weight

Review dietary characteristics				Outcomes			
		**Intervention diets**	**Control diets**	**HbA1c**	**FBG**	**FBI or non-FBI**	**Weight (wt.)**
**First author, year**	**No. of RCTs**	CHO range (% Energy or g/day)	% CHO or % total fat or low calorie - (kcal/day)	*Change (%) MD with 95% CI*	*Change (mmol/L) MD with 95% CI*	*Change (mU/L) MD with 95% CI*	*Change (Kg) MD with 95% CI Pooled data*
**Systematic review with meta-analysis *A***							
Fan, 2016	10 T2D	LC diets 20-70g/d of CHO (or 20-50% CHO of total energy (E) 1500-1800 kcal/d)	LF diets >30% energy fat (>10% SFA). HC diets (50-60% E from CHO) or usual/ ADA diet. (50-60% E from CHO of 1400-1800 kcal/d)	Significantly favours LC diets in 10 studies. -0.3% (-0.51, -0.151), P<0.001	NR	NR	Significantly favours -2.3kg (-3.65, -1.06) P<0.001
Goldenberg, 2021	23 Trials	LC diets 20-50/d of CHO, 70-130g/d, energy restricted	LF diets, iso-caloric	Significantly favours LC diets in 17 studies. 0.5% (-0.60, -0.34) P<0.001	No difference, NS in 14 studies -0.7 (-1.19, -0.27) P=NR	NR	Significantly favours in 18 studies MD -3.5kg (-5.2, -1.7) P<0.001
Huntriss, 2018	7 in MA (of 18 studies) T2D	LC diets 20-50g/day of CHO (or 20-50% energy from CHO or <130g/d)	Usual care diets 50-60% energy from CHO	Significantly favours LC diets in 7 studies analysing HbA1c. -0.3% (-0.53, -0.02) P=0.03	NR	NR	NS difference in 10 studies 0.2kg (95% CI -1.37, 1.92) P=0.7
Jamka, 2020 a	3 in MA (of 4 studies) GMD (T2D)	Paleolithic (paleo) diets 32-52% energy from CHO	HE diets or M diet, 42-52% energy from CHO	No difference, NS in 3 paleo diet studies analysing HbA1c. -0.4% (-0.87, 0.11) P=0.1	No difference, NS -0.3 (-0.87, 0.18) P=0.2	No difference, NS -0.1 mU/L (-0.6, 0.3) P=0.5	No difference, NS in 3 studies. Range of MD -0.3, to -13kg, P=NR
Korsmo-Haugen, 2019	16 in MA (of 23 studies) T2D	LC diets 20-70g/d of CHO (or 5-40% energy from CHO)	HC diets 52g/d of CHO or 40-60% energy from CHO	Favours, NS in LC diets and HE in 16 studies. MD -0.09% (-0.17, -0.01) tested # P=0.01	NR	NR	Favours, NS in 16 (+1) studies. -0.4 kg (-0.91, 0.21) P=0.07

Li, 2021	12 T2D	LC diets 20-50/d, 70-130g/d of CHO or 20% energy from CHO	LF diets, 25-30% energy from fat, 45-60% energy from CHO	Significantly favours with LC diet. MD -0.4% (95% CI -0.45, -0.24) P<0.00001	NR	NR	Significantly favours -3kg (-4.36, -1.63) P<0.00001
McArdle, 2019	25 T2D	VLCD diets, 20-50g/d to low CHO diets, (LC diet 70-130g/d to moderate CHO diets, 128-232g/d of CHO	LF diets, HC, M diet calorie-restricted diets, HE, ADA diets, Low calorie diets, LGI, or LP diets, or LF-HC: 50-60% energy from CHO	No difference, NS in pooled effect -0.09%, (-0.27, 0.08) P=0.3 VLCD: -0.1% (-0.34, 0.08) P=0.2 LC: -0.5% (-0.75, -0.23) P= 0.0002, MC: 0.1% (-0.17, 0.37) P=0.5	NR	NR	No difference, NS -0.1kg (-0.33, 0.08) P=0.2
Meng, 2017	9 T2D	LC diets 20-130g of CHO (or 5-20% energy from CHO)	HC diets or usual diets, 50-60% energy from CHO (or unclear)	Significantly favours HbA1c with LC diets -0.4% (95% CI -0.61, -0.26) P=0	No difference, NS -0.05 (-0.58, 0.47) P=0.8	NR	No difference, NS but favours in subgroup # analysis. WMD -0.9kg (95% CI -0.92, 0.05) P=0.06
Naude, 2014 #	5 include T2D (of 19 studies)	LC diets <45% energy from CHO, various energy restrictions	Wt loss diets >65% energy from CHO, and energy balance	No difference, NS in LC diet subgroup# with T2D. 3-6mo: 0.2% (-0.0, 0.39) 1-2yrs: 0.01%, (-0.28, 0.30) P=NR	NR	NR	NS difference in # T2D. 3-6mo. 5 trials MD 0.82 kg (95% CI -1.25 to 2.9) P=0.4, 1-2 yr. 4 trials MD 0.9kg, (95% CI -2.08, 3.89) P=0.6
Sainsbury, 2018	12 in analysis (of 25 studies) T2D	VLCD and LC diets ≤ 45% energy from CHO, LCD <26% E from CHO. MC diets 26-45% E from CHO	HC diets ≥45% energy from CHO, and LF, HE, ADA, LGI, M Diet or usual diet	Favours, NS reduction at 3mo. in LC, no change in MC. 3mo: -0.2% (-0.33, -0.05) P=0.008. 6mo: -0.2% (-0.31, 0.02) P=0.09. 12mo: -0.09% (-0.21, 0.03) P=0.1	NR	NR	No difference, NS in 9 studies. 6 mo: -0.1kg (95% CI -0.93, 0.65). 10 studies 12 mo: -0.1kg (95% CI -0.93, 0.07) P=NR

Snorgaard, 2017	8 in MA (of 10 studies) T2D	LC diets to moderate CHO diets 14-42% energy from CHO	HC diets 48-55% energy from CHO.	Favours, NS in LC diets lowering HbA1c short term only. 3-6mo. -0.3% (-0.63, -0.06) P=0.02. 12mo. -0.04% (-0.04, 0.13) P=0.29	NR	NR	No difference, NS in 7 studies less than 1 yr: (1.02, -1.03) over 1 yr. studies (1.36, -0.97) P=NR
van Zuuren, 2018	14 in analysis (of 36) T2D	LC diets 20-130g of CHO/d (or 10-40% energy from CHO)	LF diets 45-70% energy from CHO	Favours, NS in LC diets slightly in 14 studies. 16wks: -0.6% (-0.93, -0.17) P=0.005. 26wks: -0.3% (-0.50, -0.02) P=0.04. 2yrs: -0.2% (-0.37, -0.41) P=0.9	No difference, NS 16wks: -1.0 (-1.66, -0.28) 26wk: -0.5 (-0.91, -0.12) 2yrs: 0.4 (-1.22, 0.48) P=NR	NR	No difference, NS in 16 studies. 26wks -0.2kg (95% CI -1.65, 1.27) P=NR
Yu, 2020 b	13 T2D	HP diet, >25% energy from protein	HC diet	No difference, NS when data is pooled. MD -0.01% (95% CI -0.11, 0.10) P=0.9	No difference, NS MD -0.1 (95% CI -0.46, 0.19) P=0.43	NR	NR
**Systematic review with no meta-analysis B**							
Malaeb, 2019 b	7 RCTs (of 21 studies) T2D	HP diet 40% energy from CHO, 30% protein, 30% fat (21-30% protein consumed)	Control diet or usual diet. 55% energy from CHO, 15%-20% protein, 30% fat	No difference, NS based on 6 of 7 studies for HbA1c (range of MD 0.1% to -1.5% P=NR)	NR	NR	NR
Yamada, 2018	3 T2D	LC diet 70-180g/d energy from CHO (ER 1800 kcal)	HC diet 248g/d (ER 1800 kcal) or ideal body wt. x 25-28 kcal/d	Favours, NS in LC diets short term (range of MD -0.4% to -0.7% P=NR)	NR	NR	NR

Note. A = Systematic reviews with meta-analysis – HbA1c and wt reduction are based on meta-analysis outcomes. B = Systematic reviews (without meta-analysis) – HbA1c and wt reduction are based on statistical analysis of individual reviews. a = Paleolithic diets. b = High protein diets. d = includes type 1 diabetes (T1D).# = subgroup data. . Abbreviations: CHO = carbohydrate; ER = energy restricted; HE = healthy eating; HC = High carbohydrate; HP = high protein; kcal = kilocalorie (calorie); LC = low carbohydrate; LCMD = Low carbohydrate Mediterranean diet; LF = low fat; LGI = low glycaemic index; LP = low protein; MA = meta-analysis; MC = moderate carbohydrate; MD = mean difference; M diet = Mediterranean diet; NR = not reported; NS = not significant; SD = significant different; T1D = type 1 diabetes; T2D = type 2 diabetes; TE = total energy; VLCD = very low carbohydrate diets; WMD = weighted mean difference; wt. = weight.
