# Supplementary material for: Determining Dietary Patterns to Recommend for Type 2 Diabetes: An Umbrella Review
Source: Nutrients. 2023 Feb 8;15(4):861. doi: 10.3390/nu15040861 (PMC9958769; doi:10.3390/nu15040861)
Supplement: Supplementary file 1 [file nutrients-15-00861-s001.zip › Table S4_supp.pdf]

Supplementary Table S4 Results from reviews on glycaemic control and weight among different dietary patterns: Mediterranean versus control diets difference in HbA1c, fasting blood glucose (FBG), fasting blood insulin (FBI) and body weight

**Review dietary characteristics**				**Outcomes**			
**First author, year**	**No. of studies**	**Intervention diets**	**Control diets**	**HbA1c**	**FBG**	**FBI or non-FBI**	**Weight**
		% CHO range (% Protein, % Fat, % MUFA), s= serves	[% CHO: % Protein: % Fat]	Change (%) MD with 95% CI	Change (mmol/L) MD with 95% CI	Change (mU/L) MD with 95% CI	Change (Kg) MD with 95% CI Pooled data
			**Systematic review with meta-analysis *A***				
Esposito, 2015	3 T2D (of 8 studies with at risk, metabolic syndrome)	M diets; mostly plant based, high whole-grains, fruit, and veg	LF diet or control diet	Significantly favours M diet in meta-analyis. 3 long-term RCTs of T2D -0.5% (-0.56 to -0.38) P=0.0001.	NR	NR	No difference, NS MD=NR (range of MD -0.3 to 2.2kg) P=NR
Huo, 2015	9 RCTs	M diets, rich in fibre, vegetables, legumes, fruit, fish, MUFA, and limited red, processed meat and animal fats. [CHO: 35-55%, Protein: 15-20%, Fat: 10-40% (50% MUFA)]	HC diets, LF diets, regular diets, usual care, or American Diabetes Association. [CHO: 50-60%, Protein: 15-20%, Fat: 10-40%]	Significantly favours the M diet in MA of 9 studies. -0.3% (-0.46, -0.14) P<0.05	Significantly favours in six studies -0.7 mmol/L (-1.24, -0.21) P=NR	Favours, NS in five studies -0.55 mU/L (-0.81, -0.29) P=NR	Favours, NS in six studies -0.3kg (CI -0.55, -0.04) P=NR
Qian, 2016 c	24 T2D (of 28 studies including 4 MUFA v PUFA)	High MUFA rich diets. 10-50% energy from CHO, (14-60% protein, 30-70% fat), 17-49% MUFA	High CHO diets. 41-64% energy from CHO, (12-23% protein: 10-39% fat), 10-19% MUFA	Favours, NS in high MUFA diets. Fixed effects model -0.08% (-0.15, 0.00) or random effects model -0.11% (-0.24, 0.02) P=NR	Significantly favours in 22 studies. -0.6 mmol/L (-0.76, -0.39) or -0.6 (-0.76, -0.39) P<0.05	No difference, NS in 11 studies. -0.6 mU/L (-1.41, 0.26) P=NR	Significantly favours in 16 studies. -1.6kg (-2.89, -0.23) P<0.05
			**Systematic review with no meta-analysis *B***				

Sleiman, 2015	7 RCTs (4 RCTs MDiet for T2D, 3 T2D subgroup)	M diets; rich in fruit & veg, breads, cereals, olive oil as fat (high MUFA), low to mod fish, poultry, alcohol & little red meat.	Low fat diet or usual diet	Favours, NS in M diet. End point results: 4 studies -0.3% P = 0.012 to -0.6% P<0.01, 2 studies NR, 1 study NS	Favours, NS in 4 studies, 2 NR, 1 NS change P=NR	NR	NR

Note. A = Systematic reviews with meta-analysis – HbA1c and wt reduction are based on meta-analysis outcomes. B = Systematic reviews (without meta-analysis) – HbA1c and wt reduction are based on statistical analysis of individual reviews. c = monounsaturated fatty acid (MUFA) diets. d = includes type 1 diabetes (T1D). # = subgroup data, ^ = 12mo data. Abbreviations: Cal = calorie; CHO = carbohydrate; ER = energy restricted; HE = healthy eating; HCD = High carbohydrate diet; HPD = high protein diet; LC = Low-carbohydrate; LCMD = Low-carbohydrate; LF = low fat; LGI = low glycaemic index; MC = moderate carbohydrate; MD = mean difference; M diet = Mediterranean diet; MUFA = monounsaturated fatty acids; NR = not reported, NS = not significant, Paleo = Paleolithic diet, RCT = randomised control trial, SD = significant different; T2D = type 2 diabetes;TE = total energy; veg = vegetables; WMD = weighted mean difference; wt. = weight.*
