# Supplementary material for: Determining Dietary Patterns to Recommend for Type 2 Diabetes: An Umbrella Review
Source: Nutrients. 2023 Feb 8;15(4):861. doi: 10.3390/nu15040861 (PMC9958769; doi:10.3390/nu15040861)
Supplement: Supplementary file 1 [file nutrients-15-00861-s001.zip › Table S5_supp.pdf]

Supplementary Table S5 Results from reviews on glycaemic control and weight among different dietary patterns: Plant-based versus control diets difference in HbA1c, fasting blood glucose (FBG), fasting blood insulin (FBI) and body weight

**Review dietary characteristics**				**Outcomes**			
		**Intervention diet**	**Control diets**	**HbA1c**	**FBG**	**FBI or non-FBI** *Change*	**Weight**
**First author, year**	**No. of studies**	% CHO range (% Protein: % Fat)	[% CHO: % Protein % Fat]	*Change (%) MD with 95% CI*	*Change (mmol/L) MD with 95% CI*	*(mU/L) MD with 95% CI*	*Change (Kg) MD with 95% CI Pooled data*
**Systematic review with meta-analysis *A***							
Viguiliouk, 2015 d	9 in HbA1c analysis (of 13) 7 T2D & 2 T1D	Plant-based protein diet (soy, almond, pulses or mixed). 40-70% energy from CHO (9-30% protein, 20-40% fat)	Animal-based protein diet (casein, milk, cheese, red meat or mixed) 40-70% energy from CHO (9-30% protein, 20-40% fat)	Significantly favours in 7 studies. T2D: -0.2%, (-0.26, -0.03). P= 0.009. 2 studies T1D: MD -0.6% (95% CI -1.43, -0.15) P=0.1 Total: -0.2%, (95% CI -0.26, -0.05) P=0.005	Significantly favours. Total: -0.5 mmol/L (-0.92, -0.13) P=0.009	Significantly favours. T2D -1.5 mU/L (-2.49, -0.41) P=0.006	NR
Viguiliouk, 2019 d	9 RCTs including T2D & T1D	Vegetarian protein, LF vegan, vegetarian, or vegan diets. 49-78% energy from CHO (12-17% Protein, 11-34% fat)	Animal protein diets as LF diet, ADA, EASD, KDA or portion control or usual diets. 47-65% energy from CHO (16-22% protein, 19-37% fat]	Significantly favours vegetarian diets in 8 trials. MD -0.3% (-0.45, -0.12), SMD -1.2% (-1.89, -0.50) P=0.0006	Significantly favours in 6 trials. MD -0.6 mmol/L, (-0.99, -0.13), SMD -1.0mmol/L (-1.84, -0.24) P=0.01	NR but 1 study favours. -1.1 mU/L (-4.02, 1.74), P=0.44	Significantly favours in 6 trials. -2.2kg (-2.95, -1.34) P<0.00001
Yokoyama, 2014	6 studies T2D	LF vegan, vegan, or lacto-ovo low-protein	Omnivorous, ADA, standard diabetes diet, or LF diet	Significantly favours vegetarian diets. -0.4% (-0.62 to -0.15), P=0.001	No difference, NS. -0.4% (-1.04 to 0.32) P=0.3	NR	NR
**Systematic reviews with no meta-analysis *B***							
Toumpanakis, 2018	8 T2D studies in analysis (of 13)	LF plant-based, vegan or LF vegan	Omnivorous, KDA, EASD, IADD or LF diets.	Favours, NS. plant-based diets, 8 studies reduced HbA1c. -0.4% (analysis in 2 studies MD -0.9% P=0.002)	NR	NR	NR

Johannesen, 2020	3 T2D (in analysis of 9 studies)	LF plant-based, vegan, LF, Low calorie lacto-ovo vegetarian, LF vegan or LGI vegan	Omnivorous, KDA, Low calorie, M diet, ADA or AHA	No difference, NS. Only 1 study including T2D flavours a difference in HbA1c. P=NR	No difference, NS. P=NR	NR	NR

Note. A = Systematic reviews with meta-analysis – HbA1c and wt. reduction are based on meta-analysis outcomes. B = Systematic reviews (without meta-analysis) – HbA1c and wt. reduction are based on statistical analysis of individual reviews. d = includes type 1 diabetes (T1D). # = subgroup data. ^ = 12mo data. Abbreviations: ADA; American Diabetes Association; AHA = American Heart Association; CHO = carbohydrate; EASD = European Association for the study of diabetes guidelines; HE = healthy eating; HC = high carbohydrate; HPD = high protein diet; IADD = Italian Association of Doctors of Diabetology guidelines; KDA = Korean Diabetes Association; LC = low-carbohydrate; LF = Low fat; LGI = low glycaemic Index; MD = mean difference; M = Mediterranean; NR = not reported; NS = not significant; T2D = type 2 diabetes; TE = total energy; WMD = weighted mean difference; wt. = weight.
