# Supplementary material for: Determining Dietary Patterns to Recommend for Type 2 Diabetes: An Umbrella Review
Source: Nutrients. 2023 Feb 8;15(4):861. doi: 10.3390/nu15040861 (PMC9958769; doi:10.3390/nu15040861)
Supplement: Supplementary file 1 [file nutrients-15-00861-s001.zip › Table S6_supp.pdf]

Supplementary Table S6 Results from reviews on glycaemic control and weight among different dietary patterns: Low-Glycaemic versus control diets difference in HbA1c, fasting blood glucose (FBG), fasting blood insulin (FBI) and body weight

**Review dietary characteristics**				**Outcomes**			
		**Intervention diet**	**Control diets**	**HbA1c**	**FBG**	**FBI or non-FBI**	**Weight**
**First author, year**	**No. of studies**	% CHO range (GI score)	[% CHO: % Protein % Fat]	*Change (%) MD with 95% CI*	*Change (mmol/L) MD with 95% CI*	*Change (mU/L) MD with 95% CI*	*Change (Kg) MD with 95% CI Pooled data*
			**Systematic review with meta-analysis *A***				
Ojo, 2019	6 in MA (of 9 RCTS) T2D	LGI diet or LGI legume diet	Higher-GI diet (GI ≥ 70 for high-GI diet) and/or control diet.	Favours, NS. LGI diets in 2 studies -0.5% (-0.61 to -0.39) of 6 studies.	Significantly favours in 4 MA studies. P>0.05	NR	NR
Zafar, 2019	54 (21 RCTs T2D)	LGI diets effects on glycaemic control.	High GI / fibre diets, healthy diets, LF/ HF diets, diabetes diets	Significantly favours LGI diets in Studies 14 to 26. Type 2 diabetes in 21 studies # SMD -0.2% (-0.3, -0.09) P=NR	Significantly favours in 26 studies SMD -0.2 mmol/L (-0.27, -0.07) P=NR	No difference, NS in 14 studies SMD -0.03 mmol/L (-0.21, -0.15) P=NR	No difference, NS in 24 studies SMD 0.1 kg (-0.19, -0.01) P=NR

Note. A = Systematic reviews with meta-analysis – HbA1c and wt. reduction are based on meta-analysis outcomes. B = Systematic reviews (without meta-analysis) – HbA1c and wt. reduction are based on statistical analysis of individual reviews. # = subgroup data. Abbreviations: GI = glycaemic Index; HF = high fat; LF = low fat; LGI = low-glycaemic Index; MD = mean difference; NR = not reported; NS = not significant; T2D = type 2 diabetes; WMD = weighted mean difference; wt. = weight.
