# Supplementary material for: Determining Dietary Patterns to Recommend for Type 2 Diabetes: An Umbrella Review
Source: Nutrients. 2023 Feb 8;15(4):861. doi: 10.3390/nu15040861 (PMC9958769; doi:10.3390/nu15040861)
Supplement: Supplementary file 1 [file nutrients-15-00861-s001.zip › Table S7_supp.pdf]

Supplementary Table S7 Results from reviews on glycaemic control and weight among different dietary patterns: Multi-intervention versus control difference in HbA1c, fasting blood glucose (FBG), fasting blood insulin (FBI) and body weight

**Review dietary characteristics**				**Outcomes**			
	**No. of studies**	**Intervention diets**	**Control diets**	**HbA1c**	**FBG**	**FBI or non-FBI** *Change*	**Weight**
**First author, year**	Population	CHO range (% Energy or g/day)	[% CHO: % Protein % Fat]	*Change (%)* MD with 95% CI	*Change (mmol/L) MD with 95% CI*	*(mU/L) MD with 95% CI*	*Change (Kg) MD with 95% CI Pooled data*
			**Systematic review with meta-analysis *A***				
Ajala, 2013	16 in analysis (of 20 RCTs) T2D	Various diets included:	Control diets included:	All diets significantly favour HbA1c outcomes:	NR	NR	Various Outcomes:
		LC diets, 13%-60% E from CHO (or 20-120g CHO/ d)	LF diets, 44-55% E from CHO or 190-230g CHO	8 LC diets, -0.12% (-0.24, -0.00) P=0.04			LC diets: -0.7kg, (-1.77, 0.39) P=0.2
		HP diets (HPD), 45-45% E from CHO	LP diets, 48-55% E from CHO	2 HP diets: -0.28%, (-0.38, -0.18) P<0.00001			HP diets: 0.4kg (-0.96, 1.84) P=0.5
		M diets, Vegetarian or vegan diets, 60-75% energy from CHO (or MDiet food score)	Control diets (LF, or conventional), 60-70% energy from CHO (or M diet food score)	3 M diets: -0.5%, (-0.64, 0.30) P<0.00001			M diets: -1.8kg (2.54, -1.15) P<0.00001
		LGI diets, High-fiber diets, 37-50% E from CHO	High-GI diet, or ADA diet, 38-50% E from CHO	3 LGI diets: -0.1%, (-0.23%, -0.03) P=0.008			LGI diets: 1.4kg (-0.58, 4.36,) P=0.4
			**Systematic review with no meta-analysis B**				
Emadian, 2015	11 RCTs T2D over wt./ obese	Various interventions, all % energy (E) from CHO: LF, 55-60%; High MUFA, 45%; LF, NR; LC M diet, LGI 35%; LF vegan, 75%; LCMD, 50%; ADA, 60-70%; Wt. Mx, 55%; HPD, 40%	Various control diets, all % energy (E) from CHO: LC, 20%; HC, 60%; GL, NR; ADA, 50%; ADA, 60-70%; LF, NR; LGI, NR; Fibre modified, 45-55%; HCD, 55%	All diets favour, NS. HbA1c outcomes: MD range -0.05%, P=0.4 to -0.7%, P=0.01	NR	NR	All favour: MD ranges -0.06kg, P= 0.8 to -3.1kg, P=0.3

Papamichou, 2019	20 RCTs T2D	Various interventions, all % energy from CHO: 9 LC, 14-35%; 2 LC M diet, 35%; 1 LC or LF wt. loss, 60%; 1 Atkins, wt loss (25-40g CHO); 3 HPD, 40%; 9 LC, 14-35%; 2 LC M diet, 35%; 1 LC or LF wt. loss, 60%; 1 Atkins, wt loss (25-40g CHO); 3 HP, 40%; 1 M diet lifestyle; 1 Vegan, 75%; 1 Ma-Pi , 67%; 1 Vegetarian diet, 60%	Various control diets, all % energy from CHO: LF diet, LGI, high GI, 42-60%; TM or ADA, 50-55%; Usual Care, 55%; ADA (1200-1550 kcal/d); LPD, 49-55%; Usual care; Conventional, 60-70%; Control, 50%; Conventional, 50%	Various HbA1c outcomes: 12 LC studies 2 of 12 favours NS; 2 LC M diet studies Significantly favour; 1 LC wt. loss favours NS; 1 Atkins NS difference; 3 HP NS difference; 1 M diet favours NS; 1 vegan favours NS; 1 Ma-Pi diet favours NS; 1 Vegetarian NS difference; All diets P=NR	NR	NR	Various favour, NS: 2 LC; 2 LC M diet; 1 Vegetarian; P=NR

Note. A = Systematic reviews with meta-analysis – HbA1c and wt. reduction are based on meta-analysis outcomes. B = Systematic reviews (without meta-analysis) – HbA1c and wt. reduction are based on statistical analysis of individual reviews. # = subgroup data. ^ = 12mo data. Abbreviations: ADA = American Diabetes Association; CD = control diet; CHO = carbohydrate; DASH = dietary approaches to stop hypertension; DIG = dietary intervention group; E = energy; ER = energy restricted; GI = glycaemic index; GL = glycaemic load; HC = high carbohydrate; HP = high protein diet; LC = low-carbohydrate; LF = low fat; LP = low protein; Ma-Pi = macrobiotic vegan diet; M diet= Mediterranean diet; MC = moderate carbohydrate; MD = median difference; MUFA = monounsaturated fatty acids; NR = not reported; PUFA = polyunsaturated fatty acids; SD = standard deviation; S = significant difference; SMD = standardised mean difference; T1D = type 1 diabetes; T2D = type 2 diabetes; TM = traditional Mediterranean; vs = versus; WMD = weighted mean difference; wt. = weight.
