# Supplementary material for: Determining Dietary Patterns to Recommend for Type 2 Diabetes: An Umbrella Review
Source: Nutrients. 2023 Feb 8;15(4):861. doi: 10.3390/nu15040861 (PMC9958769; doi:10.3390/nu15040861)
Supplement: Supplementary file 1 [file nutrients-15-00861-s001.zip › Table S8_supp.pdf]

Supplementary Table S8 Results from reviews on cardiovascular markers among different dietary patterns: Dietary interventions versus control diets difference in cardiovascular outcome

First author, year (reference)	Systolic blood pressure (SBP, mm/Hg)	Diastolic blood pressure (DBP, mm/Hg)	Total cholesterol (TC, mmol/L)	Low density lipoprotein (LDL-C, mmol/L)	High density lipoprotein (HDL-C, mmol/L)	Triglycerides (TG, mmol/L)
**Low-carbohydrate diets – Systematic review with meta-analysis A**						
Fan, 2016	NR	NR	No difference, NS. WMD -0.05 mmol/L (95% CI -0.14, 0.24) P=0.6	No difference, NS. WMD -0.03 mmol/L (95% CI -0.10, 0.05) P=0.5	Significantly favours, WMD 0.094 mmol/L (95% CI 0.04, 0.14) P<0.001	Significantly favours, WMD -0.28 mmol/L (95% CI 0.39, -0.16) P<0.001
Goldenberg, 2021	NR	NR	No difference, NS. MD -0.1 (95% CI -0.41, 0.20) P=NR	No difference, NS. MD -0.02 (95% CI -0.09, 0.12) P=NR	No difference, NS. MD -0.06 (95% CI -0.01, 0.10) P=NR	No difference, NS. MD -0.3 (95% CI -0.43, 0.17) P=NR
Huntriss, 2018	Significantly favours, MD -2.74 mm/Hg (95% CI -5.27, -0.20) P=0.03	No difference, NS. MD -0.99 mm/Hg (95% CI -2.24, 0.25) P=0.1	Favours, NS. LCD MD -0.08 mmol/L (95% CI -0.23, 0.08) P=0.4	No difference, NS. MD 0.05 mmol/L (95% CI -0.10, 0.19) P=0.5	Significantly favours, MD 0.06 mmol/L (95% CI 0.04, 0.09) P=0.00001	Significantly favours, MD -0.24 mmol/L (95% CI (-0.35, -0.13) P=0.0001
Korsmo-Haugen, 2018	No difference, NS. MD -0.9 mm/Hg (95% CI -2.24, 0.37) P=0.4	No difference, NS. MD -0.2 mm/Hg (95% CI -1.20, 0.79) P=0.65	No difference, NS. MD 0.04 mmol/L (95% CI -0.12, 0.20) P=0.5	No difference, NS. MD 0.01 mmol/L (95% CI -0.13, 0.11) P=0.4	No difference, NS. MD 0.04 mmol/L (95% CI -0.01, 0.10) P=0.1	Favours, NS. LCD MD -0.1 mmol/L (95% CI -0.24, -0.02) P=0.5
Li, 2021	No difference, NS. MD 0.8 (95% CI -0.03, 0.25) P=0.6	No difference, NS. MD 0.2 (95% CI -1.71, 2.17) P=8.2	No difference, NS. MD -0.1 (95% CI -0.03, 0.25) P=0.1	No difference, NS. MD -0.03 (95% CI -0.06, 0.12) P=0.5	Significantly favours, MD 0.1 (95% CI 0.05, 0.13) P=0.00001	Significantly favours, MD -0.2 (95% CI -0.31, 0.10) P=0.0001
Meng, 2017	NR	NR	No difference, NS. WMD 0.06 mmol/L (95% CI -0.08, 0.21) P=0.6	No difference, NS. WMD -0.04 mmol/L (95% CI -0.08, 0.16) P=1.0	Significantly favours, WMD 0.07 mmol/L (95% CI 0.03, 0.11), P=0	No difference, NS. WMD -0.3 mmol/L (95% CI -0.45, -0.21) P=0.7
Naude, 2014 #	No difference, NS. 3-6 mo. MD 0.6 mm/Hg, 1-2yrs. MD 0.31 mm/Hg, P=NR	No difference, NS. 3-6 mo. MD 0.8 mm/Hg, 1-2yrs. MD 0.09 mm/Hg, P=NR	No difference, NS. 3-6 mo. MD 0.04 mmol/L, 1-2yrs. MD 0.1 mmol/L, P=NR	No difference, NS. 3-6 mo. MD 0.06 mmol/L, 1-2yrs. MD 0.1 mmol/L, P=NR	No difference, NS. 3-6 mo. MD 0.01 mmol/L, 1-2yrs. MD 0.08-0.09 mmol/L, P=NR	No difference, NS. 3-6 mo. MD 0.2 mmol/L, 1-2yrs. MD 0.08 mmol/L, P=NR

Sainsbury, 2018	No difference, NS. MD -0.2 to -16.6 mm/Hg, P= º unclear	No difference, NS. MD -0.93 to -10.0 mm/Hg, P= º unclear	No difference, NS. 3 mo. MD ranges 0.05 to -0.1 mmol/L, 12 mo. MD ranges 0.2 to -0.1 mmol/L, P= º unclear	No difference, NS. 3 mo. MD ranges 0.2 to -2.0 mmol/L, 12 mo. MD ranges 0.2 to -0.3 mmol/L, P= º unclear	No difference, NS. 3 mo. MD ranges 0.9 to 0 mmol/L, 12 mo. MD ranges 0.2 to -0.03 mmol/L, P= ^{0}unclear	No difference, NS. 3 mo. MD ranges -0.02 to -0.8, 12 mo. MD ranges -0.3 to -0.7 mmol/L, P= º unclear
Snorgaard, 2017	NR	NR	NR	No difference, NS. < 1 yr. MD 0.04 (0.13 to -0.06) more than yr. MD -0.01mmol/L (-0.01 to -0.07) P=NR	NR	NR
van Zuuren, 2018	No difference, NS. MD 8wk -2.0 mm/Hg (95% CI -25.29, 11.29) 2yrs, 0.8 mm/Hg (-3.68, 5.21) P=NR	No difference, NS. MD 8wk 5.0 mm/Hg (-1.67, 11.67) 2yrs, 0.08 (95% CI 2yrs. 0.08 mm/Hg (95% CI -2.56, 2.39) P=NR	NR	No difference, NS. MD 8wk -0.07 mmol/L (-0.41, 0.27) P = 0.7, 2yrs 0.06 mmol/L (-0.08, 0.21) P=0.4	Favours, NS. MD 8wk 0.1 mmol/L (0.00 to 0.25) P=0.05, 2yrs. 0.1mmol/L (0.07, 0.17) P<0.00001	Favours, NS. MD 8wk -0.3 mmol/L (-0.76 to 0.14) P=0.2, 2yrs. -0.2 mmol/L (-0.32, -0.05) P=0.007#
Yu, 2019 b	No difference, NS. MD -0.6 mm/Hg, (95% CI -2.45, 1.31) P=0.55	No difference, NS. MD -0.7 mm/Hg, (95% CI -2.48, 1.02) P=0.4	Significantly favours, MD -0.2 mmol/L (95% CI -0.31, -0.12), P=0.00001	Significantly favours, MD -0.1 mmol/L (95% CI -0.18, -0.02), P=0.02	No difference, NS. MD 0.03 mmol/L (95% CI -0.04, 0.11), P=0.4	Significantly favours, MD -0.2 mmol/L (95% CI -0.33, -0.05), P=0.007
**Mediterranean diets – Systematic review with meta-analysis *A***						
Huo, 2015	Favours, NS. MSD -0.5 mmHg (95% CI -1.91, -0.94) P=NR	Favours, NS. MSD -1.4 mmHg, (95% CI -1.84, -0.97) P=NR	Significantly favours, MD -0.1 mmol/L (95% CI -0.19, -0.09) P=NR	No difference, NS. MD -0.1 mmol/l (95% CI -0.24, 0.01) P=NR	Favours, NS. MD 0.06 mmol/L (95% CI 0.02, 0.10) P=NR	Significantly favours, MD -0.3 mmol/L (95% CI -0.47, -0.10) P=NR
Quan, 2016 c	Significantly favours, WMD -2.3 mmHg (95% CI -4.13, -0.49) P<0.05	No difference, NS. WMD -2.6 mmHg (95% CI -5.92, 0.63) P =NR	NR	No difference, NS. WMD 0.05 mmol/L (95% CI -0.07, 0.16) P=NR	Significantly favours, WMD 0.06 mmol/L (95% CI 0.02, 0.10) P<0.05	Significantly favours, WMD -0.3 mmol/L (95% CI -0.44, -0.18) P<0.05
**Vegetarian or plant-based diets – Systematic review with meta-analysis *A***						
Viguiliouk, 2019 d	No difference, NS. SMD 0.1 mm/Hg (95% CI -2.33, 2.52) P=0.2	No difference, NS. SMD 0.5 mm/Hg (95% CI -0.50, 1.57) P=0.5	NR	Significantly favours, SMD -0.1 mmol/L (95% CI -0.20, -0.04] P=0.002	No difference, NS. SMD -0.03 mmol/L (95% CI -0.08, 0.02) P=0.2	No difference, NS. SMD 0.1 mmol/L (95% CI -0.10, 0.38) P=0.3
**Low-glycaemic Index diets – Systematic review with meta-analysis *A***						

Zafar, 2019 #	NR	NR	No sig difference in 25 studies, SMD -0.12 mmol/L (95% CI -0.25, -0.00) P=NR	No sig difference in 23 studies, SMD -0.13 mmol/L (95% CI -0.23, -0.02) P=NR	No sig difference 23 studies, SMD -0.01 mmol/L (95% CI -0.09, -0.12) P=NR	No sig difference in 25 studies, SMD -0.09 mmol/L (95% CI -0.22, -0.04) P=NR
**Multiple diets – Systematic review with meta-analysis *A***						
Ajala 2013 e	NR	NR	NR	LC diet: No difference, NS. WMD -0.03 (95% CI -0.12, 0.07) P = 0.57 (HPD: no difference WMD -0.2 mmol/L (95% CI -0.41, 0.09) P=0.2	LC diet: Significantly, favours, WMD 0.08 (95% CI 0.05, 0.11) P<0.00001, (HPD: no difference, WMD 0.01mmol/L (95% CI -0.08, 0.10) P=0.9	LC diet: No difference, NS. WMD -0.04 (95% CI -0.15, 0.07) P=0.47, (HPD: no difference WMD -0.1 mmol/L (95% CI -0.56, 0.33) P=0.61
				M diet: No difference, NS. WMD -0.08 mmol/L (95% CI -0.24, 0.08) P=0.3	M diet: Significantly favours, WMD 0.04 mmol/L (95% CI 0.01, 0.07) P=0.004	M diet: Significantly favours, WMD -0.2 mmol/L (95% CI -0.29, -0.14) P<0.00001)
				LGI diet: No difference, NS. WMD -0.07 mmol/L (95% CI -0.16, 0.02) P=0.2	LGI diet: Significantly favours, WMD 0.05 mmol/L (95% CI 0.02, 0.07) P<0.0001	LGI diet: No difference, NS. WMD -0.07 mmol/L (95% CI -0.16, 0.02) P=0.2

Note. Review studies not included have limited or no records of cardiovascular measures. A = Systematic reviews with meta-analysis – HbA1c and wt reduction are based on meta-analysis outcomes. a = Paleolithic diets. b = High protein diets. c = Monounsaturated fatty acid (MUFA). d = includes type 1 diabetes (T1D). e = sytematic reviews that compare multiple dietary patterns are incorporated. # = subgroup data. ^ = 12mo data. º unclear = range of P values mainly not reported or not significant. Abbreviations: CHO = carbohydrate; HPD = high protein diet; LC = Low-carbohydrate; LGI = low glycaemic index; M diet = Mediterranean diet; mo. = month; NR = not reported; NS = not significant; SD = standard deviation; SMD = standardised mean difference; T1D = type 1 diabetes; T2D = type 2 diabetes; WMD = weighted mean difference.
