# Supplementary material for: Determining Dietary Patterns to Recommend for Type 2 Diabetes: An Umbrella Review
Source: Nutrients. 2023 Feb 8;15(4):861. doi: 10.3390/nu15040861 (PMC9958769; doi:10.3390/nu15040861)
Supplement: Supplementary file 1 [file nutrients-15-00861-s001.zip › Table S9_supp.pdf]

Supplementary Table S9 Conclusions and quality appraisal of dietary patterns for diabetes

Review first author	Rating methodology	Conclusions, identified bias and limitations by authors	Quality	Risk of bias
**Low-carbohydrate diet (LCD) reviews**				
**Systematic review with meta-analysis *A***				
Fan, 2016	Jadad scale. Two reviewers using Stata software for MA	Significant decrease in HbA1c with LC diets. All were more effective for weight loss than control groups, four studies showed greater results.	Moderate	Unclear
Goldenberg, 2021 #	Cochrane risk of bias tool RoB 2.0. GRADE Assessment. Sensitivity ratings with NutriGRADE.	Moderate to low certainty for diabetes remission with LCDs. Minimal clinically important differences. Safety concerns long term and confounding of calorie restrictions.	Moderate	Unclear

Huntriss, 2018	Cochrane bias risk tool used to assess.	Improvements with LC diets including weight loss, HbA1c reduced in seven studies. Adherence to VLCD a concern but LC diet achievable.	Low	Unclear
Jamka, 2020 a	Cochrane tool. Comprehensive MA software. Three independent reviewers.	Paleolithic diets in three studies did not differ from other types of healthy diets with effect on HbA1c. No difference in wt. loss for Paleo versus control diets.	Moderate	High
Korsmo-Haugen, 2018	Cochrane tool. GRADE Assessment. Reviewed by two authors.	LC diets related to overall HbA1c reduction. A range of dietary patterns are suitable, including M diet.	Low	Unclear

Li, 2021	Cochrane risk of bias tool RoB 2.0. Stata software for regression test. Two reviewers.	LC diets can improve HbA1c and weight. Practical constraints and medication changes not considered. Outcomes founded by weight change. Renal safety is uncertain.	Moderate	Unclear
McArdle, 2019	Cochrane handbook PRISMA statement. PROSPERO published protocol.	No overall pooled effect on HbA1c for restricting CHO. Significant change in HbA1c for short-term subgroup trials to 6 months.	Low	Unclear
Meng, 2017	Modified Jadad scale, scored 0 to 7 (>4 high quality). Two authors.	Significant effect on glucose control (HbA1c), positive effect on TG and HDL but not long-term wt. loss.	High	Unclear

Naude, 2014 #	Cochrane Collaboration tool. Two authors. GRADE for quality.	Little or no changes in HbA1c and CVD risk between diets at 3–6 mo. and 1–2 yrs. No mean difference in weight loss with LC diets. Adherence problematic.	Low	Unclear
Sainsbury, 2017	Cochrane handbook tool and two reviewers.	CHO restricted diets had greater reduction in HbA1c at 3-6 months. Subgroup of LC diet <26% total energy from CHO favours wt. loss.	Low	Low
Snorgaard, 2017	AMSTAR quality tool. Covidence & AGREE II software. MA using PICO. Two authors.	LC diets have greater effect on glycemic control, reducing HbA1c in first year. The greater CHO restriction, the more HbA1c lowered.	Moderate	Unclear

van Zuuren, 2018	Cochrane risk tool. Two authors assessed risk. CCTs used ROBINS-I. GRADE Ax.	CHO reduction favours glycemic control slightly but with uncertain clinical importance. Compliance issues.	Low	Unclear
Yu, 2020 b	Cochrane handbook for systematic reviews.	High protein diets do not significantly improve glycemic control or blood pressure but can lower blood lipid profiles.	Low	Low
**Systematic review with no meta-analysis *B***				
Malaeb, 2019 b	Cochrane handbook and two authors. Rayyan tool, Oxford EB-medicine.	High protein diet has no consistent benefit or negative effects. Insufficient evidence for plant vs. animal protein. Compliance and drop out issues.	Low	Unclear
Yamada, 2018	Assessed risk of bias with (Minds) Medical Information Network Distribution Service.	CHO restricted diet not energy restricted diet, may have short term glycaemic benefits.	Moderate	Low
**Mediterranean diet (MDiet) reviews**				

Review author	Rating methodology	Conclusions, identified bias and limitations by authors	Quality	Risk of bias
**Systematic review with meta-analysis *A***				
Esposito, 2015	Cochrane tool. Two reviewers and GRADE.	M diets associated with better glycaemic control and reduced CVD risk factors than control diets. Analysis of three long-term trials showed significant difference.	Moderate	Low
Huo, 2015	Cochrane Collaborators tool. PRISMA reporting. Two independent investigators.	Mediterranean style diets improve outcomes for glycaemic control, body weight and CVD risk.	High	Low

Qian, 2016 c	Cochrane Collaborators tool. Two independent reviewers.	High MUFA diets improve glycaemic control and metabolic risk factors with reduced plasma glucose, lipids, and wt. loss. Long- and short-term studies.	Moderate	Low
**Systematic review with no meta-analysis *B***				
Sleiman, 2015	Quality tool NR	Most studies show favorable effects of the MDiet on glycemic control and CVD	Low	Unclear

Plant-based diet reviews

**Review author**	**Rating methodology**	**Conclusions, identified bias and limitations by authors**	**Quality**	**Risk of bias**
**Systematic review with meta-analysis *A***				
Viguiliouk, 2015 d	Cochrane risk tool. Heyland Methodological Quality Score (MQS).	Overall replacing animal with plant protein leads to significant modest increases in glycemic control.	Low	Unclear

Viguiliouk, 2018 d	Cochrane risk tool. GRADE approach. Two investigators.	Vegetarian diets significantly lower HbA1c, FBG, FBI, weight and LDL-cholesterol but have no effect on other blood lipids and blood pressure.	Moderate	Unclear
Yokoyama, 2014	Cochrane handbook criteria for systematic reviews.	Vegetarian diets associated with significant reduction in HbA1c compared to control diets, greater in two non-randomised studies.	Low	Unclear
**Systematic reviews with no meta-analysis *B***				
Johannesen, 2020	McMaster University quality Ax tool. Two researchers.	Plant-based diet favorable for glycemic control with T2D &/or obesity. one of three RCTs significantly improve glycemic control. Compliance issues.	Low	Unclear

Toumpanakis, 2018	Quality tool NR. PRISMA checklist and flowchart.	Plant-based diets with educational interventions can significantly improve psychological health, QOL, HbA1c and weight. Adherence has greater HbA1c reduction.	Low	Unclear

Low Glycaemic Index (LGI) diet reviews

**Review author**	**Rating methodology**	**Conclusions, identified bias and limitations by authors**	**Quality**	**Risk of bias**
**Systematic review with meta-analysis** ***A***				
Ojo, 2019	Critical Appraisal Skills Program Evaluation checklist. PICOS criteria.	LGI diets more effective in controlling HbA1c and FBG compared with higher-GI or control diets for T2D.	Moderate	Low
Zafar, 2019 #	Cochrane Collaboration tool. Two reviewers.	LGI diet may be useful for glycaemic control compared to other diet types. May not be effective in achieving wt. loss in people with type 2 diabetes.	Low	High

Multiple intervention diet reviews

Review author	Rating methodology	Conclusions, identified bias and limitations by authors	Quality	Risk of bias
**Systematic review with meta-analysis *A***				
Ajala, 2013	Cochrane Collaboration tool. PRISMA guidelines. Cohort-type using ROBINS-1	LC, LGI, M diet and HP diets all lead to significantly improved glycemic control compared with control. Largest HbA1c effect size was M diet. Wt. loss favours M diet, not significant in LC, LGI, no benefit in HPD.	High	Unclear
**Systematic review with no meta-analysis *B***				
de Carvalho, 2020	Cochrane tool for RCTs and crossover studies. ROBINS-I for cohort studies.	Dietary patterns vegan/ vegetarian/ M diet/ DASH all favoured glycemic control, in reduction of HbA1c. Most diets studied were vegan or vegetarian patterns.	Moderate	Unclear

Emadian, 2015	JBI critical appraisal tool. Two reviewers.	4 of 11 trials show promise in improving HbA1c levels including LC-M diet, LF-Vegan, LGI diets, independent of wt loss.	Low	Unclear
Papamichou, 2019	Cochrane risk of bias tool.	No significant change in glycemic control or wt in LC diets. Vegan, macrobiotic diets improved HbA1c. M diet showed reduction in HbA1c, wt. and delayed medications. Vegetarian diet reduced wt.	Moderate	High

Note. The Cochrane Collaboration assessment tool was used to rate each individual review for biases. A = Systematic reviews with meta-analysis – HbA1c and wt reduction are based on meta-analysis outcomes. B = Systematic reviews (without meta-analysis) – HbA1c and wt reduction are based on statistical analysis of individual reviews. a = Paleolithic diets. b = High protein diets. c = Monounsaturated fatty acid (MUFA). d = includes type 1 diabetes (T1D). # = subgroup data. Abbreviations: Cal = calorie; CHO = carbohydrate; Cochrane = a global independent network of researchers; CVD = cardiovascular disease; ER = energy restricted; GRADE = grading approach for quality of evidence and strength of recommendations; HE = healthy eating; HCP = High carbohydrate diet; HPD = high protein diet; JBI = Joanna Briggs Institute; LC = Low-carbohydrate; LCMD = Low-carbohydrate Mediterranean diet; LF = low fat; LGI = Low-glycaemic index; MCD = moderate carbohydrate diet; MD = mean difference; M diet = Mediterranean diet; MUFA = monounsaturated fatty acids; NR = not reported; NS = not significant; Paleo = Paleolithic diet; RCT = randomised control trial; PICO = population, intervention, comparator, and outcomes; PRISMA = statement for reporting SR and MA of studies that evaluate health care interventions; SD = significant different; T2D = type 2 diabetes; TE = total energy; veg = vegetables; WMD = weighted mean difference; wt. = weight.
